# Family history, obesity, urological factors and diabetic medications and their associations with risk of prostate cancer diagnosis in a large prospective study

**DOI:** 10.1038/s41416-022-01827-1

**Published:** 2022-05-24

**Authors:** Visalini Nair-Shalliker, Albert Bang, Sam Egger, Xue Qin Yu, Karen Chiam, Julia Steinberg, Manish I. Patel, Emily Banks, Dianne L. O’Connell, Bruce K. Armstrong, David P. Smith

**Affiliations:** 1 The Daffodil Centre, The University of Sydney, a joint venture with Cancer Council NSW, Sydney, New South Wales (NSW) Australia; 2 Faculty of Medicine and Health Sciences, Macquarie University, Sydney, NSW Australia; 3 Specialty of Surgery, Sydney Medical School, The University of Sydney and Department of Urology, Westmead Hospital, Sydney, NSW Australia; 4 National Centre for Epidemiology and Population Health, Australian National University, Canberra, Australian Capital Territory Australia; 5 School of Medicine and Public Health, University of Newcastle, Newcastle, NSW Australia; 6 School of Population and Global Health, University of Western Australia, Perth, Western Australia Australia; 7 School of Public Health and Preventive Medicine, Monash University, Melbourne, Victoria Australia

**Keywords:** Risk factors, Cancer epidemiology

## Abstract

**Background:**

Prostate cancer (PC) aetiology is unclear. PC risk was examined in relation to several factors in a large population-based prospective study.

**Methods:**

Male participants were from Sax Institute’s 45 and Up Study (Australia) recruited between 2006 and 2009. Questionnaire and linked administrative health data from the Centre for Health Record Linkage and Services Australia were used to identify incident PC, healthcare utilisations, Prostate Specific Antigen (PSA) testing reimbursements and dispensing of metformin and benign prostatic hyperplasia (BPH) prescriptions. Multivariable Cox and Joint Cox regression analyses were used to examine associations by cancer spread, adjusting for various confounders.

**Results:**

Of 107,706 eligible men, 4257 developed incident PC up to end 2013. Risk of PC diagnosis increased with: PC family history (versus no family history of cancer; HR_adjusted_ = 1.36; 95% CI:1.21–1.52); father and brother(s) diagnosed with PC (versus cancer-free family history; HR_adjusted_ = 2.20; 95% CI:1.61–2.99); severe lower-urinary-tract symptoms (versus mild; HR_adjusted_ = 1.77; 95% CI:1.53–2.04) and vasectomy (versus none; HR_adjusted_ = 1.08; 95% CI:1.00–1.16). PC risk decreased with dispensed prescriptions (versus none) for BPH (HR_adjusted_ = 0.76; 95% CI:0.69–0.85) and metformin (HR_adjusted_ = 0.57; 95% CI:0.48–0.68). Advanced PC risk increased with vasectomy (HR_adjusted_ = 1.28; 95% CI:1.06–1.55) and being obese (versus normal weight; HR_adjusted_ = 1.31; 95% CI:1.01–1.69).

**Conclusion:**

Vasectomy and obesity are associated with an increased risk of advanced PC. The reduced risk of localised and advanced PC associated with BPH, and diabetes prescriptions warrants investigation.

## Introduction

Prostate cancer (PC) is the second most common cancer diagnosed in men worldwide and the most common cancer diagnosed in men from developed countries [1]. Australia has the highest incidence of PC internationally and, in 2021, an estimated 18,000 new PC cases will be diagnosed in Australia.

Despite its high incidence, there is limited evidence on risk factors for PC. Established risk factors include advancing age, African ancestry and family history of PC [2–6]. All these factors are non-modifiable, thus providing no basis for primary prevention of PC. While a diagnosis of cutaneous melanoma and obesity are associated with PC incidence, only the latter is potentially modifiable [7, 8]. There is emerging evidence that pharmaceuticals prescribed for the treatment of diabetes are related to the risk of a range of malignancies including PC [9–15]. Results from two large population-based prospective studies showed a reduced risk of incident PC in men taking pharmaceuticals for diabetes [16, 17]. It is suggested that the anti-tumour effects of prescriptions for diabetes may be due to their anti-proliferative effects.

Some urological factors are associated with PC risk including prescriptions for benign prostatic hyperplasia (BPH), lower-urinary-tract symptoms (LUTS) and vasectomy. These associations, especially with BPH prescriptions, vasectomy and LUTs may be at least partly attributable to increased medical surveillance [18–21]. The PSA test, the most commonly used biomarker in prostate cancer surveillance, was introduced in Australia in the late-1980s and the rise in its uptake corresponded with a rapid increase in PC incidence [22]. Thus, when seeking possible PC risk factors, the potential confounding effects of PSA testing on their associations with PC must be considered.

The current study prospectively examined potential risk factors for PC diagnosis in the 45 and Up Study, a large Australian population-based study of 267,153 male and female participants recruited in New South Wales (NSW). The study’s aim was to assess whether selected sociodemographic, behavioural and health-related factors were associated with a higher or lower risk of PC diagnosis, while accounting for possible confounding by PSA testing.

## Methods

### Study population

The Sax Institute’s 45 and Up Study is a large NSW population-based cohort study of male and female participants aged 45 years and above [23]. All participants were enrolled between 2006 and 2009. Participants were randomly sampled from Services Australia (formerly the Australian Government Department of Human Services) Medicare enrolment database: Services Australia is a publicly funded universal healthcare system that covers all citizens, permanent residents and some temporary residents and refugees. Those aged over 80 years and residents of regional areas were over-sampled by a factor of two. All participants completed a postal questionnaire at recruitment, which included information on sociodemographic factors, health behaviours and medical history and provided consent for linkage of their data to selected population health databases.

The 45 and Up Study was approved by the University of NSW Human Research Ethics Committee, while approval for this analysis of PC risk factors was given by the NSW Population and Health Services Research Ethics Committee (HREC/14/CIPHS/54). The use of Services Australia’s Medicare Benefits Schedule (MBS) and Pharmaceutical Benefits Scheme (PBS) data was approved by the Australian Department of Health’s Departmental Ethics Committee.

### Record linkage

In 2017, the Centre for Health Record Linkage (CHeReL) linked records of all participants with selected administrative health records. These records were NSW Cancer Registry (NSWCR: January 1994–December 2013), Cause of Death Unit Record File (CODURF: February 2006–December 2015), Registry of Births, Deaths and Marriages records (RBDM: February 2006–December 2016) and NSW Admitted Patient Data Collection (APDC: July 2001–June 2016). All pathology laboratories, hospitals and radiotherapy and medical oncology departments are required by the Public Health Act 2010 to report all newly diagnosed cancers to the NSWCR. Information on the degree of spread at diagnosis is assigned within 4 months of PC diagnosis. However, if not all clinical information is received within this period of data acquisition, then these cases are classified as ‘Unknown’ spread [24, 25]. Probabilistic record linkage with these administrative health databases provided information on participants’ cancer diagnoses, procedures during admissions to public and private hospitals and death. In addition, the Sax Institute used a unique identifier to link study data deterministically with records of claims made to the Medicare Benefits Schedule (MBS: Sep 2005–Dec 2016) and Pharmaceutical Benefits Scheme (PBS: Sep 2005–Dec 2016) provided by Services Australia. This linkage provided information on PSA tests reimbursed by Medicare, General Practitioner (GP) consultations and relevant medications dispensed.

### Exclusion criteria

**Fig. 1:**
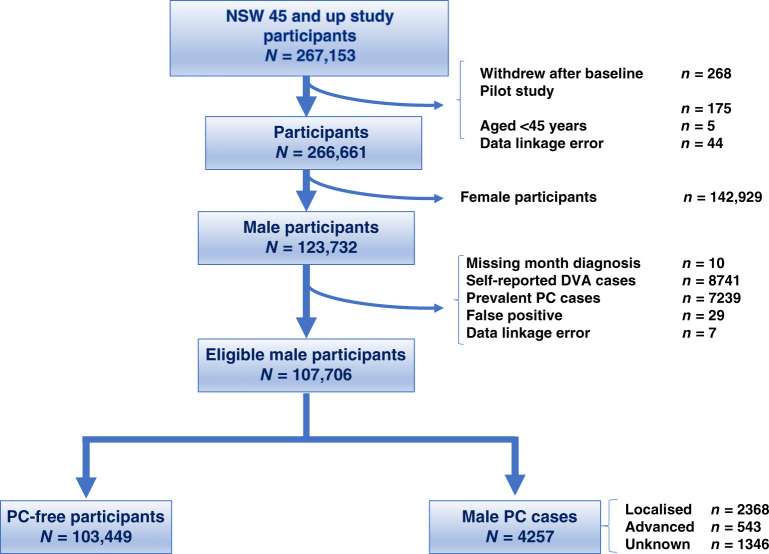
Selection of 45 and Up Study participants for analysis. Participants with a PC diagnosis prior to recruitment to the Study, participants with errors in linkage records and Department of Veterans’ Affairs (DVA) cardholders, were excluded.

1. PC diagnosis prior to recruitment to the 45 and Up Study (illustrated in Fig. 1). These were men with a record for PC diagnosis (ICD-10 C61) registered by the NSWCR, with APDC records listing C61 diagnosis codes or a radical prostatectomy recorded in Medicare Benefits Schedule (MBS) claims.
2. Participants with linkage errors in NSWCR, CODURF or RBDM records.
3. Department of Veterans’ Affairs (DVA) cardholders were excluded because a proportion of their medical and pharmaceutical benefits are funded by the Department of Veterans Affairs and these data were not available. DVA clients were identified via self-report or any mention of DVA coverage in hospital or emergency department records.

### Exposure variables

The following PC risk factors of primary interest to this analysis were derived using self-reported information at recruitment:

family history of cancer (history of only PC, history of prostate and breast and/or ovarian cancers, history of other cancer(s) and no family history of cancer [reference]); family history of PC (father only, brother only, father and brother and no family history of PC [reference]); body mass index (BMI in kg/m^2^) based on height (m) and weight (kg) and categorised according to the World Health Organisation classification for normal (<24.9 kg/m^2^), overweight (25.0–29.9 kg/m^2^) or obese (≥30 kg/m^2^); [26] height divided into quartiles; weekly alcohol consumption categorised as non-drinkers with the remaining values divided into quartiles; physical activity from reported number of vigorous and non-vigorous physical activity sessions in a normal week consolidated and categorised as 0–3, 4–6, 7–10, 10–17, 18+ sessions per week; [27] LUTS measured using the modified International Prostate Symptom Score (m-IPSS) and categorised as having no, mild (0–5), moderate (6–11) and severe symptoms (12–21); [28] erectile dysfunction (ED) categorised as men who reported they can always or usually get and keep an erection firm enough for satisfactory sexual activity (No) and men who could not (Yes); smoking (Ever, Never) and vasectomy (Yes, No).

The following variables were obtained by linkage with PBS, MBS and APDC records up to 3 years before PC diagnosis or the censoring date: dispensing of medications to treat BPH (Anatomical Therapeutic Classification (ATC) code C02CA01, C02CA04, D11AX10 and G04C), to treat diabetes using metformin (PBS ATC code A10BA02) or non-metformin medications (PBS ATC code beginning with A10 but excluding A10BA02) with subsidised measurement of glycosylated haemoglobin (MBS 66551) or APDC code indicating diabetes contributed to need for a hospitalisation. Those with missing information for a single variable were included in the analysis as a “missing” category.

### Outcome variable

PC cases were defined as men with a record for ICD-10 code C61 cancer registered with the NSWCR after enrolment in the 45 and Up Study and before the end of follow-up on 31 December 2013 (the last date for which data used in these analyses was available).

### Potential confounders

Potential confounders available in the current cohort study based on previous publications were identified and referenced [2, 5, 6].

All participants’ and, separately, PC cases’ sociodemographic, lifestyle and behavioural characteristics, as self-reported at the time of recruitment, and relevant health-related factors obtained by linkage to administrative records are listed in Table 1. The place of residence classified according to the Accessibility Remoteness Index of Australia (ARIA+) was derived from the residential postcode. The following variables were obtained by linkage to MBS and APDC for which data were available from June 2004: Charlson Comorbidity Index (CCI) for non-cancer comorbidities was measured using a score derived from diagnosis codes in hospital admissions records up to 3-year period prior to PC diagnosis or date of censoring; uptake of PSA testing was based on MBS claims made from September 2005 until the date of PC diagnosis (or censoring date) for the MBS items for PSA screening (#66655) and monitoring (#66656 or #66659 or #66660), calculated as a rate per 5 years and grouped as having no record of PSA testing or monitoring (reference category), having a record for PSA monitoring but not PSA testing and having PSA testing at varying rates; frequency of MBS reimbursed visits to a general practitioner (GP) was calculated as a number of visits per 5 years and categorised into quartiles with 0–<12 visits as the reference category (Table 2).

**Table 1:** Characteristics of NSW 45 and Up Study male participants without a prostate cancer diagnosis and those diagnosed with prostate cancer between study entry and December 2013.

Characteristics	Participants^a^		PC cases	
	*n*	%^b^	*n*	%^b^
**Total**	103,449	96.0%	4257	4.0%
**Demographic characteristics**				
Age (years)				
45–64	63,973	61.8	1916	45.0
65–79	30,943	29.9	1965	46.2
80+	8533	8.2	376	8.8
Median age at recruitment, years (min, max)	61.0	(45.0, 102.8)	65.9	(45.8, 96.7)
Married or living with partner	83,072	80.3	3484	81.8
Born in Australia/NZ	77,242	74.7	3300	77.5
Private health insurance	57,282	55.4	2307	54.2
Living in major cities	53,890	52.1	2168	50.9
Annual Household income $70,000+	30,220	29.2	922	21.7
University degree	26,219	25.3	967	22.7
Median rate PSA testing # tests/5 years (min, max)^c^	0.9	(0, 8.6)	1.10	(0.10.5)
Median rate PSA monitoring # tests/5 years (min, max)^c^	0.6	(0, 26.4)	2.44	(0, 28.2)
Median rate of GP visits # visits/5 years (min, max)^d^	24.6	(0, 524.0)	28.42	(0, 240.4)
**Physical, behavioural and other health-related factors**				
Family history of prostate cancer	6208	6.0	349	8.2
Median height, cm (min, max)	176.5	(55.0, 241.3)	176.5	(65.0, 226.1)
Median weight, kg (min, max)	83.0	(34.9, 255.0)	82.0	(40.0, 220.0)
Median BMI, kg/m^2^ (min, max)	26.8	(15.0, 50.0)	26.6	(15.1, 48.5)
Ever smokers	52,743	51.0	2101	49.4
Median weekly alcohol consumption, # drinks/week (min, max)	6	(0, 40)	7	(0, 100)
Median #weekly sessions physical activity (min, max)	10	(0, 1530)	10	(0, 455)
No comorbidities^e^	85,917	83.1	3725	87.5
Self-reported vasectomy	26,051	25.2	1076	25.3
Self-reported erectile dysfunction	30,651	29.6	1584	37.2
Self-reported severe LUTS	2761	2.7	206	4.8
Prescription for BPH^f^	8790	8.5	423	9.9
Diabetes prescription-only Metformin^f^	5082	4.9	138	3.2
Diabetes prescription-only non-Metformin^f^	2045	2.0	66	1.6
Diabetes prescription-Mix of Metformin, and non-Metformin^f^	16,083	15.5	548	12.9

^a^Excluding PC cases.

^b^% for ‘total’ is row% while other % are column %.

^c^MBS records for PSA testing (MBS code 66655) and monitoring (MBS code 66656, 66659, 66660) before PC diagnosis or censoring date.

^d^GP visits from MBS records before PC diagnosis or censoring date.

^e^Based on Charlton comorbidity index for MBS records before PC diagnosis or censoring date.

^f^Prescription information from PBS records before PC diagnosis or censoring date.

**Table 2:** Hazard ratios (HR) and 95% CI for diagnosis of prostate cancer and sociodemographic and health-related characteristics for NSW 45 and Up Study male participants (*n* = 107,706).

Characteristics	Person years	No PC *n* = 103,449	PC *n* = 4257	HR^a^	95% CI		HR^b^	95% CI			HR^c^	95% CI		
Region of birth														
Australia or New Zealand	444,916	77,242	3300	1.00			1.00				1.00			
Other countries	149,729	26,207	957	0.80	0.74	0.86	0.81		0.75	0.87	0.84		0.78	0.91
* p*-value^d^					<0.0001				<0.0001				<0.0001	
Health cover														
None	100,164	17,393	534	1.00			1.00				1.00			
Healthcare concession card	149,443	26,676	1329	1.11	1.00	1.23	1.07		0.97	1.19	1.07		0.96	1.19
Private health insurance	333,391	57,282	2307	1.19	1.09	1.31	1.11		1.01	1.23	1.10		0.99	1.21
Missing	11,647	2098	87	1.08	0.86	1.36	1.11		0.89	1.40	1.12		0.89	1.41
* p*-value^d^					0.0006				0.11				0.20	
Income														
Less than $19,999 per year	103,647	18,608	939	1.00			1.00				1.00			
$20,000–$29,999 per year	56,121	9763	507	1.00	0.90	1.12	0.93		0.83	1.04	0.92		0.82	1.03
$30,000–$39,999 per year	48,896	8399	392	0.97	0.86	1.10	0.87		0.77	0.98	0.85		0.75	0.96
$40,000–$49,999 per year	47,308	8139	342	0.99	0.87	1.12	0.87		0.76	0.99	0.85		0.74	0.97
$50,000–$69,999 per year	70,737	12,059	481	1.05	0.94	1.17	0.91		0.80	1.02	0.88		0.78	0.99
$70,000 or more per year	175,903	30,220	922	1.01	0.91	1.11	0.86		0.76	0.96	0.83		0.74	0.94
Missing	92,031	16,261	674	0.91	0.83	1.01	0.85		0.77	0.95	0.87		0.78	0.96
* p*-value^d^					0.93				0.12				0.028	
Qualification														
No school certificate	61,964	11,008	486	1.00			1.00				1.00			
School or intermediate certificate	86,777	15,188	702	1.10	0.98	1.23	1.05		0.93	1.18	1.04		0.92	1.17
Higher school or leaving certificate	57,158	9933	389	1.06	0.93	1.21	1.06		0.92	1.21	1.05		0.92	1.21
Trade or apprenticeship	113,004	19,710	816	1.04	0.93	1.16	1.01		0.90	1.13	0.99		0.88	1.11
Certificate or diploma	113,944	19,661	818	1.10	0.98	1.23	1.05		0.94	1.18	1.03		0.92	1.16
University degree or higher	152320	26,219	967	1.06	0.95	1.18	1.02		0.91	1.15	0.99		0.88	1.12
Missing	9476	1730	79	1.06	0.83	1.34	1.11		0.87	1.41	1.12		0.88	1.43
* p*-value^d^					0.60				0.90				0.79	
Place of residence														
Major cities	309,667	53,890	2168	1.00			1.00				1.00			
Inner regional	206,807	35,945	1517	1.05	0.98	1.12	1.04		0.98	1.11	1.04		0.97	1.11
Outer regional / remote	66,546	11,588	496	1.06	0.96	1.17	1.06		0.96	1.16	1.05		0.95	1.16
Missing	11,625	2026	76	0.95	0.76	1.20	0.95		0.75	1.19	0.95		0.75	1.19
* p*-value^d^					0.2714				0.3472				0.392	
Marital status														
Single / widowed divorced / separated	108,199	19,396	730	1.00			1.00				1.00			
Married / living with partner	481,250	83,072	3484	1.07	0.99	1.16	1.01		0.93	1.10	1.00		0.92	1.09
Missing	5196	981	43	1.27	0.93	1.73	1.28		0.94	1.74	1.28		0.94	1.75
*p*-value^d^					0.086				0.79				0.98	
Comorbidity^e^														
0	503,878	85,917	3725	1.00			1.00				1.00			
1	44,857	8126	268	0.63	0.55	0.71	0.64		0.56	0.72	0.70		0.61	0.79
2+	45,910	9406	264	0.57	0.50	0.64	0.58		0.51	0.66	0.67		0.59	0.77
* p*-value^d^					<0.0001				<0.0001				<0.0001	
PSA testing rate PSA^f^ tests per 5 years														
No record of PSA testing or monitoring	62,822	11,767	95	1.00			1.00				1.00			
No record of PSA testing + 1 monitoring^g^	71,281	12,285	1068	7.31	5.93	9.03	7.24		5.86	8.95	6.99		5.66	8.64
<1 PSA testing per 5 years	163,091	27,869	827	2.78	2.25	3.44	2.75		2.22	3.40	2.70		2.18	3.34
1 to <2 PSA testing per 5 years	221,566	38,359	1348	3.16	2.56	3.89	3.09		2.51	3.81	3.02		2.45	3.73
2 to <3 PSA testing per 5 years	4800	8309	615	6.18	4.98	7.68	6.00		4.82	7.46	5.85		4.70	7.28
3+ PSA testing	27,876	4860	304	5.00	3.97	6.31	4.78		3.79	6.03	4.66		3.69	5.89
*p*-value^d^					<0.0001				<0.0001				<0.0001	
Frequency GP visits^h^ per 5 years														
0–14.4	153,179	26,383	691	1.00			1.00				1.00			
14.4 < 25.21	148,921.	25,439	1136	1.25	1.14	1.38	1.13		1.03	1.25	1.14		1.03	1.25
25.21 < 41.95	150,085	25,906	1224	1.11	1.00	1.22	1.00		0.91	1.11	1.02		0.93	1.13
41.95 < 523.98	142,459	25,721	1206	1.02	0.92	1.13	0.98		0.88	1.09	1.02		0.92	1.14
* p*-value^d^					<0.0001				0.0039				0.016	

^a^Adjusted for age (as underlying time variable).

^b^Adjusted for age (as underlying time variable), region of birth, health cover, income, qualification, place of residence, marital status, Charlson’s comorbidity index unless variable is the exposure of interest, frequency of PSA testing and frequency of GP visits.

^c^Additionally adjusted for family history of cancer, BMI, smoking alcohol, physical activity, lower-urinary-tract symptoms, vasectomy, erectile dysfunction, medications for BPH and medications for diabetes.

^d^*P*-values are based on tests that exclude the HRs for missing value categories.

^e^Based on Charlson’s comorbidity index for APDC records before PC diagnosis or censoring date.

^f^MBS records for PSA testing (MBS code 66655) and monitoring (MBS code 66656, 66659, 66660) before PC diagnosis or censoring date.

^g^Men with no record for PSA testing (MBS code 66655) but have at least one record for monitoring (MBS code 66656, 66659, 66660).

^h^MBS records for primary healthcare visits before PC diagnosis or censoring date.

### Statistical analyses

Hazard ratios (HR) and 95% confidence intervals (CI) were estimated using Cox Proportional Hazards regression, with age as the underlying time variable, using SAS version 9 (SAS Institute Inc., Cary, NC, US). In these analyses, the endpoint was PC diagnosis or with censoring on the earlier of December 31, 2013, date death or the diagnosis date of cancer other than PC. Joint Cox Proportional Hazards regression, with age as the underlying time variable, was used to distinguish time to diagnosis based on the spread of disease defined as localised or advanced (regional and metastatic spread). Men with missing information to determine disease spread are classified as ‘unknown’ were excluded from the main Joint Cox regression analysis. The regression analyses were, at a minimum, adjusted for age, region of birth, health cover, income, qualifications, place of residence, marital status, Charlson comorbidity index, frequency of PSA testing, frequency of primary healthcare visits and fully adjusted for family history of cancer, BMI, smoking alcohol, physical activity, lower-urinary-tract symptoms, vasectomy, erectile dysfunction, medications for BPH and medications for diabetes (as listed in footnotes of each table).

Sensitivity analysis excluding the first year of follow-up for all participants after the date of enrolment in the 45 and Up Study was carried out to reduce the possible impact of reverse causation. Although our analysis examined incident PC in an era where PSA testing was widespread, we conducted a second sensitivity analysis to determine the impact of excluding a highly screened group. National Australian clinical practice guidelines for PSA testing and early management of test-detected PC, recommend men who have been informed of the benefits and harms of testing and who decide to undergo regular testing for prostate cancer, should be offered PSA testing every 2 years from age 50–69 [29]. Based on this, participants who had 3 or more PSA tests in the 5 years before the end of follow-up were defined as highly screened participants and were excluded.

## Results

From the 267,153 participants in the 45 and Up Study, participants were excluded if they withdrew from the study after baseline (*n* = 268), were participants from the pilot study (*n* = 175), were aged below 45 years (*n* = 5) or were female (*n* = 142,929). Of the remaining 123,732 male participants, there were 107,706 eligible men for our analysis (Fig. 1). Of these, 4257 men were diagnosed with PC during the study period.

The median time between recruitment to study and PC diagnosis was 2.89 years. The median age of PC cases at recruitment was 65.9 years, 77.5% of men were born in Australia or New Zealand, 54.2% had private insurance cover, 50.9% were living in major cities and 81.8% reported being married or living with a partner (Table 1). Among PC cases, 8.2% reported having a family history of PC, had a median BMI of 26.6 kg/m^2^, 49.4% reported ever having smoked cigarettes, drank a median of 7 alcoholic drinks per week and did 10 h of median physical activity per week. Prior to PC diagnosis, there were 3.2% of PC cases identified as having a dispensed record for PBS subsidised prescription for metformin, and 9.9% of cases with prescription records for BPH treatment. Participants’ characteristics are summarised in Table 1. Sociodemographic and behavioural factors associated with increased risk of PC diagnosis were being born in Australia or New Zealand, and having private health cover, low income, no comorbidities, increased frequency of PSA testing and frequent visits to GP (Table 2).

**Fig. 2:**
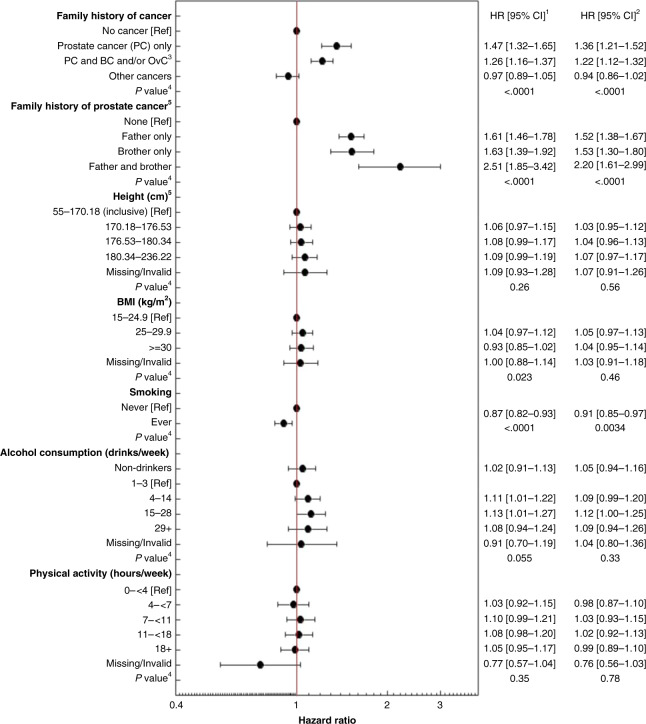
Hazard ratios (HR) and 95% confidence intervals (CI) of prostate cancer diagnoses (to 2013) in the 45 and Up Study. Risk for prostate cancer diagnosis in categories of personal and behavioural characteristics in male Study participants (*n* = 107,706).

Multivariable regression analyses in a fully adjusted model showed that the overall risk of PC diagnosis was highest in men with a family history of PC (HR_adjusted_ = 1.36; 95% CI 1.21–1.52; Fig. 2), and in men with a brother and father diagnosed with PC (HR_adjusted_ = 2.20; 95% CI 1.61–2.99). The risk of PC diagnosis was slightly lower in ever versus never smokers (HR_adjusted_ = 0.91; 95% CI 0.85–0.97), while height, BMI, alcohol consumption and physical activity showed no association with PC diagnosis.

**Fig. 3:**
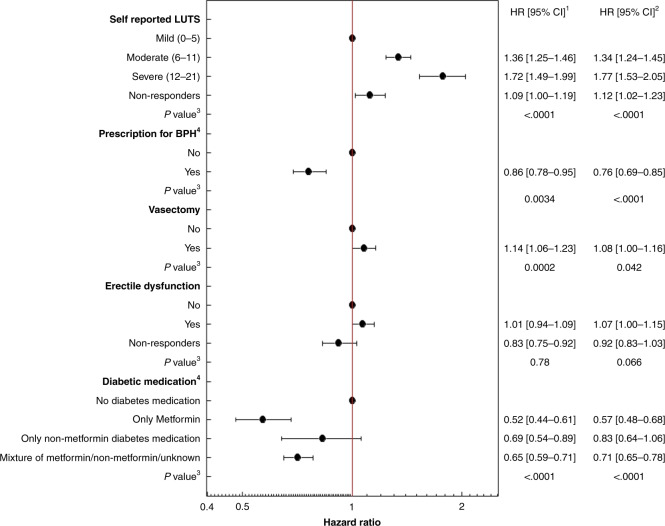
Hazard ratios (HR) and 95% confidence intervals (CI) of prostate cancer diagnoses (to 2013) in the 45 and Up Study. Risk of prostate cancer diagnosis in categories of health-related factors in male Study participants (*n* = 107,706).

Analysis of urological factors showed an increased risk of PC diagnosis associated with increased severity of LUTS (Fig. 3; HR_adjusted_ = 1.77; 95% CI: 1.53–2.05 for severe versus mild), and a weak association with vasectomy (HR_adjusted_ = 1.08 95% CI: 1.00–1.16 for yes versus no); there was no association with previously reporting having emergency department records. The risk of PC diagnosis was lower in men with a record for PBS subsidised prescription for BPH treatment (HR_adjusted_ = 0.76; 95% CI: 0.69–0.85 for yes versus no), and prescription for metformin (HR_adjusted_ = 0.57; 95% CI: 0.48–0.68 for yes versus no) but no association with non-metformin medications for diabetes—Fig. 3.

Joint Cox regression analysis showed some evidence that the association between PC and vasectomy, and between PC and obesity, differed according to the stage of disease (*p* = 0.052 and *p* = 0.05, respectively) Specifically, men who had a vasectomy had a higher relative risk of advanced PC (HR = 1.28 95% CI: 1.06–1.55) than localised (HR = 1.04 95% CI: 0.95–1.14). Similarly, obese men (versus normal BMI) had a higher relative risk of advanced PC (HR = 1.31 95% CI: 1.01–1.69) than localised PC (HR = 0.98 95% CI: 0.87–1.12). There was no evidence of variation by stage of disease for all other exposures examined (Table 3).

**Table 3:** Joint Cox regression (HR, 95% CI) for diagnosis of localised or advanced prostate cancer and personal, behavioural and health-related characteristics for 45 and Up Study male participants (*n* = 106,360).

Variable	PC cases (*n*)		HR^a^ (95% CI)		HR^b^ (95% CI)	
	Localised	Advanced	Localised	Advanced	Localised	Advanced
Family History of cancer						
No cancer	1287	316	1.00	1.00	1.00	1.00
Prostate cancer (PC) only	216	42	1.65 (1.43, 1.90)	1.31 (0.95, 1.81)	1.52 (1.31, 1.75)	1.20 (0.87, 1.66)
PC and BC and/or OvC^c^	433	90	1.32 (1.19, 1.48)	1.13 (0.89, 1.43)	1.28 (1.15, 1.43)	1.09 (0.86, 1.38)
Other cancers	432	95	0.96 (0.86, 1.07)	0.87 (0.69, 1.09)	0.93 (0.83, 1.04)	0.84 (0.67, 1.05)
* p*-value^d^			0.42		0.41	
Family history of prostate cancer^e^						
None	1970	462	1.00	1.00	1.00	1.00
Father only	292	57	1.81 (1.60, 2.05)	1.52 (1.15, 2.00)	1.71 (1.51, 1.93)	1.42 (1.07, 1.87)
Brother only	84	19	1.68 (1.35, 2.09)	1.62 (1.02, 2.57)	1.57 (1.26, 1.96)	1.51 (0.96, 2.40)
Father and brother	22	5	2.52 (1.66, 3.84)	2.42 (1.00, 5.84)	2.15 (1.41, 3.27)	2.20 (0.91, 5.32)
* p*-value^d^			0.72		0.69	
BMI (kg/m^2^)						
15–24.9	669	149	1.00	1.00	1.00	1.00
25–29.9	1121	233	1.06 (0.97, 1.17)	1.03 (0.84, 1.26)	1.06 (0.96, 1.17)	1.04 (0.84, 1.28)
≥30	431	113	0.90 (0.79, 1.01)	1.10 (0.86, 1.41)	0.98 (0.87, 1.12)	1.31 (1.01, 1.69)
Missing	147	48	0.98 (0.82, 1.17)	1.45 (1.05, 2.01)	1.00 (0.84, 1.20)	1.54 (1.11, 2.13)
* p*-value^d^			0.044		0.050	
Height (cm)^e^						
55–170.18 (inclusive)	593	119	1.00	1.00	1.00	1.00
170.18–176.53	592	122	1.02 (0.91, 1.15)	1.08 (0.84, 1.39)	0.99 (0.89, 1.12)	1.04 (0.81, 1.34)
176.53–180.34	625	145	1.03 (0.92, 1.16)	1.24 (0.97, 1.59)	1.00 (0.89, 1.12)	1.17 (0.92, 1.50)
180.34–241.30	466	127	1.00 (0.88, 1.13)	1.43 (1.11, 1.84)	0.98 (0.87, 1.11)	1.36 (1.06, 1.76)
Missing	92	30	1.01 (0.81, 1.25)	1.65 (1.11, 2.47)	0.98 (0.79, 1.23)	1.62 (1.08, 2.42)
* p*-value^e^			0.063		0.10	
Alcohol consumption (drinks/week)						
Non-drinkers	500	125	1.03 (0.89, 1.19)	1.20 (0.88, 1.64)	1.06 (0.92, 1.23)	1.27 (0.93, 1.73)
1–3	280	59	1.00	1.00	1.00	1.00
4–14	967	204	1.19 (1.04, 1.36)	1.19 (0.89, 1.60)	1.17 (1.03, 1.34)	1.15 (0.86, 1.54)
15–28	404	108	1.16 (1.00, 1.36)	1.51 (1.10, 2.08)	1.17 (1.00, 1.36)	1.46 (1.06, 2.02)
29+	181	41	1.12 (0.93, 1.35)	1.24 (0.83, 1.84)	1.16 (0.96, 1.40)	1.23 (0.82, 1.85)
Missing	36	6	1.06 (0.75, 1.49)	0.80 (0.35, 1.86)	1.21 (0.85, 1.71)	0.90 (0.39, 2.10)
* p*-value^d^			0.34		0.33	
Physical activity (sessions/week)						
0–<4	335	78	1.00	1.00	1.00	1.00
4–<7	339	91	0.98 (0.84, 1.14)	1.15 (0.85, 1.56)	0.93 (0.80, 1.08)	1.12 (0.83, 1.53)
7–<11	585	118	1.08 (0.95, 1.24)	0.96 (0.72, 1.28)	1.02 (0.89, 1.16)	0.92 (0.69, 1.23)
11–<18	592	125	1.08 (0.94, 1.23)	1.01 (0.76, 1.33)	1.00 (0.87, 1.15)	0.96 (0.72, 1.28)
18+	488	125	1.01 (0.88, 1.16)	1.15 (0.86, 1.52)	0.94 (0.82, 1.09)	1.08 (0.81, 1.44)
Missing	29	6	0.89 (0.61, 1.30)	0.78 (0.34, 1.80)	0.88 (0.60, 1.29)	0.77 (0.33, 1.76)
* p*-value^d^			0.24			0.27
Smoking						
Never	1204	281	1.00	1.00	1.00	1.00
Ever	1164	262	0.87 (0.80, 0.94)	0.85 (0.71, 1.00)	0.90 (0.83, 0.98)	0.89 (0.74, 1.06)
Missing				NA		
* p*-value^d^			0.78		0.86	
LUTS						
Mild (0–5)	1438	352	1.00	1.00	1.00	1.00
Moderate (6–11)	490	103	1.41 (1.27, 1.57)	1.19 (0.95, 1.49)	1.38 (1.24, 1.54)	1.23 (0.98, 1.54)
Severe (12–21)	122	21	1.90 (1.58, 2.28)	1.31 (0.84, 2.03)	1.93 (1.60, 2.34)	1.46 (0.93, 2.29)
Missing	318	67	1.07 (0.95, 1.21)	0.86 (0.66, 1.13)	1.10 (0.97, 1.24)	0.90 (0.69, 1.18)
* p*-value^d^			0.15		0.40	
Erectile dysfunction						
No	1256	305	1.00	1.00	1.00	1.00
Yes	848	183	1.05 (0.95, 1.15)	0.85 (0.70, 1.04)	1.11 (1.00, 1.22)	0.94 (0.76, 1.16)
Missing	264	55	0.86 (0.75, 0.99)	0.66 (0.49, 0.89)	0.95 (0.82, 1.10)	0.74 (0.54, 1.01)
* p*-value^d^			0.067			0.17
Vasectomy						
No	1759	386	1.00	1.00	1.00	1.00
Yes	609	157	1.11 (1.01, 1.22)	1.36 (1.12, 1.64)	1.04 (0.95, 1.14)	1.28 (1.06, 1.55)
* p*-value^d^			0.061		0.052	
Prescription for BPH^f^						
No	2143	503	1.00	1.00	1.00	1.00
Yes	225	40	0.87 (0.76, 1.00)	0.65 (0.47, 0.90)	0.76 (0.66, 0.87)	0.63 (0.45, 0.89)
*p*-value^d^			0.11		0.33	
Prescription for diabetes^f^						
None	1945	458	1.00	1.00	1.00	1.00
Only metformin	73	17	0.50 (0.39, 0.63)	0.50 (0.31, 0.81)	0.55 (0.43, 0.69)	0.56 (0.34, 0.91)
Only Non-Metformin diabetes medication	32	10	0.63 (0.44, 0.89)	0.81 (0.44, 1.52)	0.74 (0.52, 1.06)	0.99 (0.52, 1.87)
Mixture of metformin and non-metformin	318	58	0.69 (0.61, 0.78)	0.54 (0.41, 0.71)	0.75 (0.67, 0.86)	0.60 (0.45, 0.80)
* p*-value^d^			0.35		0.41	

^a^Adjusted for age as the underlying time variable.

^b^Adjusted for age, region of birth, health cover, income, qualification, place of residence, marital status, Charlson’s comorbidity index, frequency of PSA testing, frequency of GP visits, family history of cancer, BMI, smoking alcohol, physical activity, lower-urinary-tract symptoms, vasectomy, erectile dysfunction, medications for BPH and medications for diabetes, unless variable is the exposure of interest.

^c^*BC* breast cancer, *OvC* ovarian cancer.

^d^*P*-values are for tests of HR equality between PC stage excluding the HRs of missing value categories.

^e^Family history of prostate cancer was not adjusted for family history of cancer; height was not adjusted for BMI.

^f^Information obtained from PBS records before PC diagnosis or censoring date.

The sensitivity analyses excluding all participants in the first year of follow-up (Supplementary Tables 1–3; Supplementary Figs. 1–3) and excluding frequent PSA testers (Supplementary Fig. 4), did not appreciably change the estimated HR (Supplementary Tables 4,5). Joint Cox regression analysis with the inclusion of PC cases with unknown status for stage showed no significant variation in HR estimates (*p*-value > 0.05) for all exposures examined (Supplementary Table 6).

## Discussion

This study prospectively examined the evidence for several known or suspected causative or protective factors for PC. The risk of localised and advanced PC diagnosis was elevated in men with a family history of PC, multiple first-degree family members diagnosed with PC, severe LUTS, while the risk of advanced PC was increased in men who were obese or had a vasectomy. The overall PC risk was reduced in men with dispensed records for BPH medications or metformin. All analyses were adjusted for sociodemographic, lifestyle and health-related factors, as well as GP visits and frequency of PSA testing, prior to PC diagnosis.

Our findings for increased risk of being diagnosed with PC in men with a family history of PC, and a 2.5-fold risk in men with multiple first-degree family members with PC, is consistent with current evidence [3, 30–32]. PC is one of the most heritable malignancies with over 170 genetic variants identified to be associated with PC, in genome-wide association studies, which together account for 28.4% of the familial relative risk (compared to ~6% accounted for by rare genetic variants) [33, 34]. A meta-analysis of 33 epidemiological studies reported increased PC risk in men with a brother (RR = 3.14; 2.37–4.15) or father (RR = 2.35; 2.02–2.72) diagnosed with PC and this risk increased ~4-fold in men with 2 or more first-degree family members with PC (RR-4.39; 2.61–7.39). Given that family history is a combination of genetic, environmental and behavioural factors, the lower risk estimates observed in our study may in part be due to less variability in these factors due to the short follow-up period of 4–7 years since recruitment, as well as lack of information on the number of affected brothers, all of which may have biased results towards the null.

Our analysis of urological factors showed some evidence that for men who reported having a vasectomy, the relative risk of advanced PC was higher (HR_adjusted_ = 1.28; 95% CI:1.06–1.55; *p* = 0.052) than the relative the relative risk of localised PC (HR = 1.04; 95% CI: 0.95–1.14). This finding is consistent with a 24-year follow-up of the Health Professionals Follow-Up Study, which reported no association between vasectomy and localised or low-grade disease, but an increased risk of high-grade (hazard ratio [HR], 1.22; 95% CI, 1.03–1.45) and advanced-stage prostate cancer (HR, 1.20; 95% CI, 1.03–1.40) [35]. A 15-year follow-up of participants from the European Prospective Investigation into Cancer and Nutrition (EPIC) study reported no association between vasectomy and overall or advanced PC; association by tumour grade was not reported [36]. A meta-analysis of 13 cohort studies showed weak evidence for a positive association between vasectomy and PC incidence (HR = 1.08; 95% CI 1.02, 1.14), but association by grade or stage of disease was not reported [37]. The biological mechanism underlying an association between vasectomy and PC risk is unclear.

The risk of localised and advanced PC was increased with increasing severity of LUTS but decreased in men with the prescription record(s) for BPH treatment. The role of LUTS in the etiologic development of PC and BPH share much in common and thus estimating the true association between LUTS and PC risk is complicated. LUTS is a self-reported measure, defined by increased frequency of urination, incomplete emptying of the bladder or waking during the night to void. It may be caused by BPH through direct obstruction of the bladder outlet or, may be associated with PC when the developing neoplasm exerts pressure on the bladder outlet or the urethra [38]. Results from two large cohorts of men who had all undergone PSA testing, the Prostate testing for cancer and Treatment study from UK (ProtecT) and Stockholm3 study from Sweden, reported no association between LUTS and PC risk [39, 40]. However the HUNT2 study from Norway reported an increased risk of localised but not advanced PC [41]. This uncertainty may partly be due to diagnostic bias such as increased PSA testing [21]. Despite controlling for frequency of PSA testing prior to PC diagnosis, a ~2-fold increased incidence of PC in men with severe LUTS was still evident in our study.

BPH is a non-malignant enlargement of the prostate that is clinically diagnosed. Severe BPH symptoms are commonly treated with minimally invasive surgery while mild to moderate symptoms are treated with medications that can shrink the prostate and ease urination. A systematic review of 8 trials showed that 5-alpha-reductase inhibitors (5-ARIs), a class of pharmaceutical prescriptions used in the treatment of BPH reduced the risk of PC diagnosis among men who regularly screened for PC. The Prostate Cancer Prevention Trial (PCPT) and the Reduction by Dutasteride of Prostate Cancer Event trial (REDUCE) which were specifically designed to examine the impact of 5-ARIs (finasteride and dutasteride) on PC risk, reported an overall 22.5–24.8% decreased risk of low-risk PC, but an increased incidence of high-risk disease [42–44]. Similar associations were reported in a 20-year follow-up of BPH cases [45]. We were unable to categorise PC cases by disease grade; however, our findings of reduced PC risk in localised and advanced PC suggest the association may be present in localised high risk as well as advanced cases. Further investigation in men with clinically significant PC may provide a better understanding of this association.

Metformin, commonly used as first-line therapy for diabetes which is the fastest growing chronic condition in Australia, may have a role in PC prevention due to its glucose-lowering and anti-proliferative properties [9, 11, 15, 17, 46, 47]. Diabetics have reduced PSA levels and given that biopsies are conducted in response to elevated PSA levels, this may delay PC diagnosis in diabetics and consequently lead to a diagnosis of more advanced disease However our results showed that the risk of localised as well as advanced PC diagnosis was reduced in men with prescription records for metformin. There is uncertainty in the evidence for an association between metformin and PC risk, and some of these inconsistencies may partly be due to the selection of study populations [11, 48]. The Finnish Randomised Study of Screening for Prostate Cancer showed that the risk of incident PC was lower in anti-diabetic drug users versus non-users (0.81; 0.61–0.95); however, sub-group analyses within diabetics, showed no association with PC incidence [17]. ‘Non-users’ of metformin, which is the reference group used in these studies, in a general population is a group that is generally free of diabetes, whereas ‘non-users’ in a diabetic population is a group that is diagnosed with diabetes but have chosen not to take medication. As such the effect in ‘users’ compared to ‘non-users’ in diabetics may be attenuated compared to that in the general population. Analyses restricted to diabetics are consistent with that reported by most studies which may have biased results towards null [11, 16, 48]. Various in vitro and clinical studies have associated elevated circulating insulin levels with tumour growth and increased cell proliferation. Metformin has the capacity to reduce tumour proliferation in a multitude of mechanisms that include reducing circulating insulin and glucose levels as well as regulating the expression of various cell signalling pathways involved in the regulation of insulin and glucose metabolism [49]. One small clinical trial of metformin in PC cases showed that metformin distributed to human prostate tissue, suggesting that metformin could exert its effects directly on tissue targets [50]. There are a few randomised clinical trials currently underway that will determine the potential chemoprevention properties of metformin for PC [51].

Obese men in our study had a higher relative risk of advanced PC diagnosis (HR = 1.54; 95% CI: 1.11–2.13) than localised PC (HR = 1.00; 95% CI: 0.84–1.20) (*p* = 0.05 for difference between HRs) and this is consistent with previous reports [7, 8]. We have previously reported higher PSA testing rates for obese men, but our recent study of this cohort reported lower biopsy rates but a higher likelihood of positive biopsy for obese men compared to men with a normal BMI [52, 53]. There is evidence that PSA levels are inversely associated with BMI, which may delay the diagnosis of PC [54]. Also, increased PC risk may be associated with obesity in men with high-grade disease. Men classified with localised disease in our study are a heterogeneous population with low to high-risk diseases diagnosed at an early stage. This heterogeneity may have attenuated the association and biased the hazards ratio towards the null [55]. Analysis by grade of disease was not possible.

The study sample comprises approximately 10% of the total population of NSW aged 45 and above and, consistent with most cohort studies, has a relatively modest response rate of 18% [56]. However, the associations quantified here are based on internal comparisons and previous methodological work reported that such comparisons are valid even though the cohort is not fully representative of the target population [56]. Research study participants as a group are healthier than the general population and may be more engaged in testing and screening behaviours than the general population, increasing the potential risk of diagnostic bias. Adjustments for PSA testing by previous studies have compared risk in the pre- and post-PSA testing eras, adjusted for baseline PSA levels, or adjusted for self-reported PSA testing from information collected at baseline [5, 35, 57]. Our ability to adjust for the rate of PSA testing, GP visits and other health comorbidities in the period prior to PC diagnosis (or censoring) for each participant through administrative record linkage, allowed us to minimise the risk of confounding related to differential levels of medical surveillance, is a strength. Joint Cox regression analysis was used to examine the association between each risk factor and the time to diagnosis for each stage of PC. This may have minimised the risk of error measurements in the time-dependent variables, which in this case is the spread of disease. Linkage with PBS data allowed us to derive information on dispensing of medications for diabetes and BPH, in the period prior to PC diagnosis (or censoring) for each participant. A limitation is that PBS data were only available since 2002 and therefore analysis for the duration of use was not possible. Furthermore, PBS data before 2012 used in this study, do not include data for medicines costing below the general beneficiary co-payment and so information for these medications for non-concessional patients may be missing. However, a validation study by Comino et al showed that the use of reimbursement records for diabetes prescriptions for 2002–2004 had a positive predictive value of ~85% for study participants who reported having diabetes in the baseline questionnaire, and this suggests that the effect of underestimation may be minimal [58]. Our classification of localised PC cases does not separate those with clinically significant disease from those with low risk or indolent cancers due to a lack of information on clinical pathology, and this is a limitation.

Since the early 1990s, PSA testing in Australia increased substantially with corresponding increases in the diagnosis of localised cancers and a fall in the rate of advanced cancers. Increased PSA testing has been documented in higher socio-economic status groups and in those with ‘healthier’ behaviours [53]. Therefore, adjusting for PSA testing behaviour is important in understanding the risk factors in localised (more likely to be screen-detected) and advanced cancers separately. Our findings show a range of established and emerging factors were associated with the risk of a diagnosis of localised and advanced PC, after adjusting for participants’ history of PSA testing. Further research is required to determine the association of these risk factors in the diagnosis of clinically significant PC.

## Supplementary information

Supplementary Figures 1,2,3, 4

Supplementary Tables 1,2,3,5,6

Legends for Supplementary Figures and Tables

Reproducibility checklist

## Data Availability

The authors confirm that, for approved reasons, some access restrictions apply to the data underlying the findings. We obtained the data for the project from a third party, namely the Sax Institute, which is the data custodian for the 45 and Up Study. Data are available through application to the Sax Institute. Details are available at https://www.saxinstitute.org.au/our-work/45-up-study/ or through contacting 45andUp.research@saxinstitute.org.au.
