# Supplementary material for: Family history, obesity, urological factors and diabetic medications and their associations with risk of prostate cancer diagnosis in a large prospective study
Source: Br J Cancer. 2022 May 24;127(4):735–46. doi: 10.1038/s41416-022-01827-1 (PMC9381576; doi:10.1038/s41416-022-01827-1)
Supplement: Supplementary file 1 — Supplementary Figures 1,2,3, 4 [file 41416_2022_1827_MOESM1_ESM.docx]

Supplementary Figure 1 Selection of participants for the sensitivity analyses excluding the first year of follow-up after study entry for male participants in the 45 and Up Study.

Supplementary Figure 2 Associations between being diagnosed with prostate cancer and **personal and behavioural** characteristics after excluding the first year of follow-up after study entry for male participants in the 45 and Up Study (n=106,996).


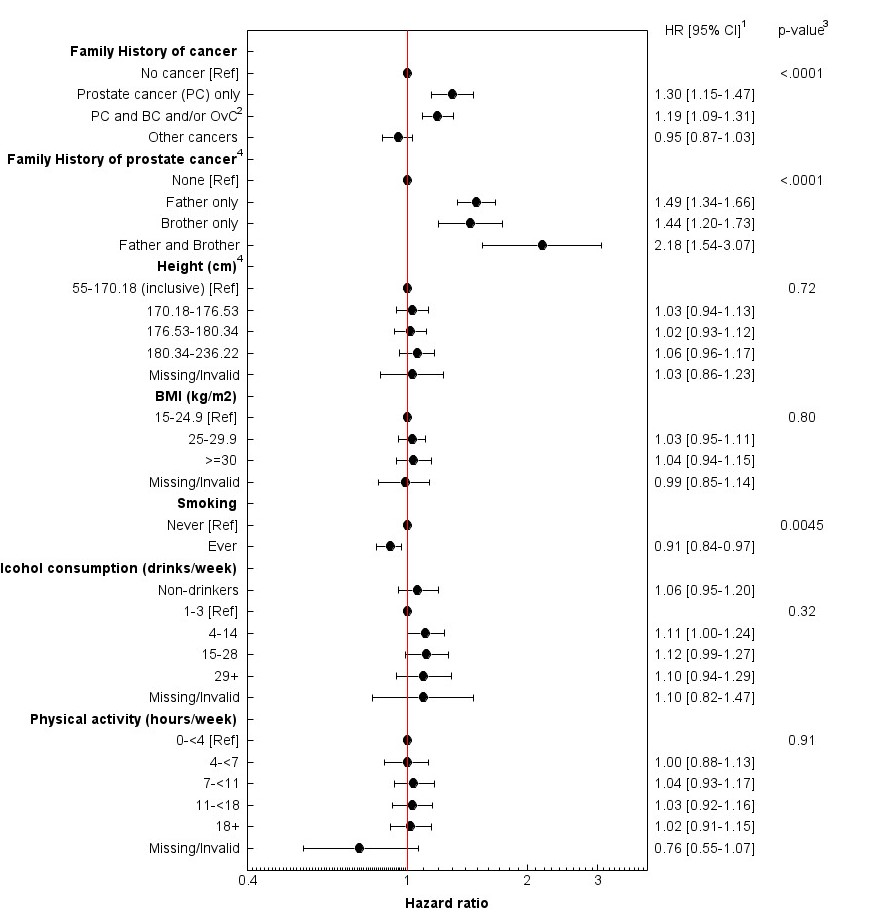


^1^Adjusted for Age, region of birth, health cover, income, qualification, place of residence, marital status, Charlson’s comorbidity index, frequency of PSA testing, frequency of GP visits, family history of cancer, BMI, smoking alcohol, physical activity, lower urinary tract symptoms, vasectomy, erectile dysfunction, prescription for BPH and prescription for diabetes, unless variable is the exposure of interest.

^2^ BC=breast cancer and OvC=ovarian cancer

^3^P-values are for tests of HR equality between PC stage excluding the HRs of missing value categories.

^4^ Family history of prostate cancer was not adjusted for family history of cancer; height was not adjusted for BMI;

Supplementary Figure 3 Associations between being diagnosed with prostate cancer and **health-related characteristics** after excluding the first year of follow-up after study entry for male participants in the 45 and Up Study (n=106,996).


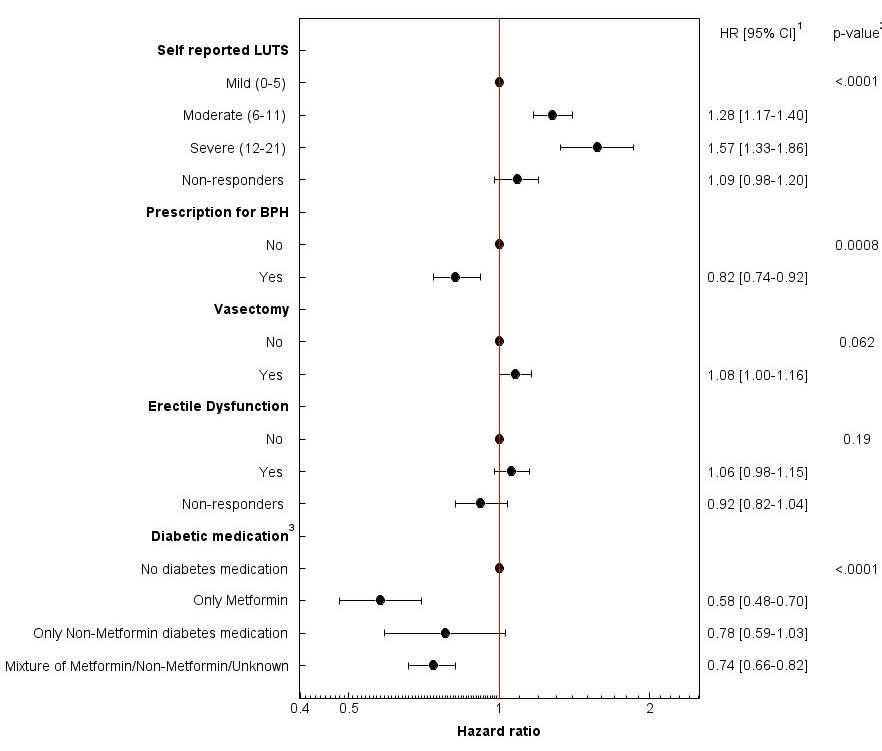


^1^Adjusted for Age, region of birth, health cover, income, qualification, place of residence, marital status, Charlson’s comorbidity index, frequency of PSA testing, frequency of primary health care visits, family history of cancer, BMI, smoking alcohol, physical activity, lower urinary tract symptoms, vasectomy, erectile dysfunction, prescription for BPH and prescription for diabetes, unless variable is the exposure of interest.

^2^P-values are for tests of HR equality between PC stage excluding the HRs of missing value categories.

^3^Information obtained from PBS and MBS records before PC diagnosis or censoring date.

Supplementary Figure 4 Selection of 45 and Up study participants excluding high PSA testers in the 45 and Up Study (n=102,542).
