# Supplementary material for: Family history, obesity, urological factors and diabetic medications and their associations with risk of prostate cancer diagnosis in a large prospective study
Source: Br J Cancer. 2022 May 24;127(4):735–46. doi: 10.1038/s41416-022-01827-1 (PMC9381576; doi:10.1038/s41416-022-01827-1)
Supplement: Supplementary file 2 — Supplementary Tables 1,2,3,5,6 [file 41416_2022_1827_MOESM2_ESM.docx]

Supplementary Table 1- Characteristics of NSW 45 and Up Study male participants and those diagnosed with prostate cancer between study entry and December 2013, after excluding the first year of follow-up after study entry for all male participants in the 45 and Up Study.

| **Characteristics** | **Participants^1^** | |  | **PC cases** | |
| --- | --- | --- | --- | --- | --- |
|  | n | %^2^ |  | n | % |
| **Total** | 103449 | 96.0 |  | 3547 | 3.3 |
| **Demographic characteristics** |  |  |  |  |  |
| Age (years) |  |  |  |  |  |
| 45-64 | 63973 | 61.8 |  | 1670 | 47.1 |
| 65-79 | 30943 | 29.9 |  | 1579 | 44.5 |
| 80+ | 8533 | 8.2 |  | 298 | 8.4 |
| Median age at recruitment, years (min, max) | 61.0 | (45.0, 102.8) |  | 65.5 | (45.8, 96.7) |
| Married or living with partner | 83072 | 80.3 |  | 2905 | 81.9 |
| Born in Australia/NZ | 77242 | 74.7 |  | 2753 | 77.6 |
| Private health insurance | 57282 | 55.4 |  | 1965 | 55.4 |
| Living in major cities | 53890 | 52.1 |  | 1801 | 50.8 |
| Annual Household income $70,000+ | 30220 | 29.2 |  | 810 | 22.8 |
| University degree | 26219 | 25.3 |  | 824 | 23.2 |
| Median rate PSA testing # tests/5 years (min, max)^3^ | 0.9 | (0, 8.6) |  | 1.03 | (0, 6.1) |
| Median rate PSA monitoring # tests/5 years (min, max)^3^ | 0.6 | (0, 26.4) |  | 2.4 | (0, 22.5) |
| Median rate of GP visits # visits/5years (min, max)^4^ | 24.6 | (0, 524.0) |  | 27.7 | (0, 240.4) |
| **Physical, behavioural and other health related factors** |  |  |  |  |  |
| Family history of prostate cancer | 6208 | 6.0 |  | 280 | 7.9 |
| Median Height, cm (min, max) | 176.5 | (55.0, 241.3) |  | 176.0 | (65.0, 226.1) |
| Median Weight, kg (min, max) | 83.0 | (34.9, 255.0) |  | 82.0 | (40.0, 220.0) |
| Median BMI, kg/m^2^ (min, max) | 26.8 | (15.0, 50.0) |  | 26.6 | (15.2, 48.5) |
| Ever smokers | 52743 | 51.0 |  | 1738 | 49.0 |
| Median weekly alcohol consumption, # drinks/week (min, max) | 6 | (0, 140) |  | 7.0 | (0, 100) |
| Median #weekly sessions physical activity (min, max) | 10 | (0,1530) |  | 10.0 | (0, 455) |
| No comorbidities^5^ | 85917 | 83.1 |  | 3104 | 87.5 |
| Self-reported Vasectomy | 26051 | 25.2 |  | 908 | 25.6 |
| Self-reported Erectile Dysfunction | 30651 | 29.6 |  | 1278 | 36.0 |
| Self-reported Severe LUTS | 2761 | 2.7 |  | 154 | 4.3 |
| Prescription for BPH^6^ | 8790 | 8.5 |  | 364 | 10.3 |
| Diabetes prescription-only Metformin^6^ | 5082 | 4.9 |  | 117 | 3.3 |
| Diabetes prescription-only non-Metformin^6^ | 2045 | 2.0 |  | 51 | 1.4 |
| Diabetes prescription-Mix of Metformin, and non-Metformin ^6^ | 16083 | 15.5 |  | 467 | 13.2 |

^1^ Excluding PC cases

^2^ % for ‘total’ is row% while other % are column %.

^3^MBS records for PSA testing (code 66655) and monitoring (code 66656, 66659, 66660) before PC diagnosis or censoring date.

^4^GP visits from MBS records before PC diagnosis or censoring date.

^5^Charlton comorbidity index for MBS records before PC diagnosis or censoring date.

^6^Prescription information from PBS records before PC diagnosis or censoring date.

Supplementary Table 2- Hazard ratios (HR) and 95% CI for diagnosis of prostate cancer and sociodemographic and health-related characteristics, after excluding the first year of follow-up after study entry for male participants in the 45 and Up Study (n=106,996).

| **Characteristics** |  | **Person years** | **No PC (n)** | **PC (n)** |  | **HR^1^ (95% CI)** |  | **HR^2^ (95% CI)** |
| --- | --- | --- | --- | --- | --- | --- | --- | --- |
| **Region of birth** |  |  |  |  |  |  |  |  |
| Australia or New Zealand |  | 444637 | 77242 | 2753 |  | 1.00 |  | 1.00 |
| Other countries |  | 149645 | 26207 | 794 |  | 0.80 (0.74,0.86) |  | 0.84 (0.77,0.91) |
| **p-value^3^** |  |  |  |  |  | <0.0001 |  | <0.0001 |
| **Health cover** |  |  |  |  |  |  |  |  |
| None |  | 100124 | 17393 | 457 |  | 1.00 |  | 1.00 |
| Health care concession card |  | 149310 | 26676 | 1060 |  | 1.06 (0.95,1.19) |  | 1.03 (0.92,1.16) |
| Private health insurance |  | 333212 | 57282 | 1965 |  | 1.19 (1.08,1.32) |  | 1.10 (0.99,1.22) |
| Missing |  | 11636 | 2098 | 65 |  | 0.97 (0.75,1.26) |  | 1.01 (0.78,1.31) |
| **p-value^3^** |  |  |  |  |  | 0.0004 |  | 0.30 |
| **Income** |  |  |  |  |  |  |  |  |
| Less than $19,999 per year |  | 103553 | 18608 | 753 |  | 1.00 |  | 1.00 |
| $20,000-$29,999 per year |  | 56073 | 9763 | 416 |  | 1.02 (0.90,1.15) |  | 0.93 (0.83,1.06) |
| $30,000-$39,999 per year |  | 48859 | 8399 | 322 |  | 0.98 (0.86,1.12) |  | 0.86 (0.75,0.98) |
| $40,000-$49,999 per year |  | 47279 | 8139 | 281 |  | 0.99 (0.86,1.14) |  | 0.85 (0.73,0.98) |
| $50,000-$69,999 per year |  | 70695 | 12059 | 402 |  | 1.05 (0.93,1.19) |  | 0.88 (0.77,1.01) |
| $70,000 or more per year |  | 175846 | 30220 | 810 |  | 1.05 (0.94,1.17) |  | 0.86 (0.75,0.98) |
| Missing |  | 91977 | 16261 | 563 |  | 0.94 (0.84, 1.05) |  | 0.89 (0.79,1.00 |
| **p-value^3^** |  |  |  |  |  | 0.87 |  | 0.13 |
| **Qualification** |  |  |  |  |  |  |  |  |
| No school certificate or other qualification |  | 61920 | 11008 | 402 |  | 1.00 |  | 1.00 |
| School or intermediate certificate |  | 86706 | 15188 | 559 |  | 1.05 (0.92,1.19) |  | 0.99 (0.87,1.13) |
| Higher school or leaving certificate |  | 57127 | 9933 | 323 |  | 1.05 (0.90,1.21) |  | 1.03 (0.88,1.19) |
| Trade or apprenticeship |  | 112941 | 19710 | 692 |  | 1.06 (0.93,1.19) |  | 1.00 (0.88,1.13) |
| Certificate or diploma |  | 113875 | 19661 | 686 |  | 1.09 (0.96,1.23) |  | 1.02 (0.89,1.15) |
| University degree or higher |  | 152247 | 26219 | 824 |  | 1.06 (0.94,1.20) |  | 0.98 (0.86,1.11) |
| Missing |  | 9466 | 1730 | 61 |  | 1.00 (0.76,1.31) |  | 1.05 (0.80,1.37) |
| **p-value^3^** |  |  |  |  |  | 0.86 |  | 0.97 |
| **Place of residence** |  |  |  |  |  |  |  |  |
| Major Cities |  | 309476 | 53890 | 1801 |  | 1.00 |  | 1.00 |
| Inner Regional |  | 206684 | 35945 | 1270 |  | 1.06 (0.98,1.14) |  | 1.05 (0.98,1.13) |
| Outer Regional, Remote, very Remote |  | 66503 | 11588 | 412 |  | 1.06 (0.95,1.18) |  | 1.06 (0.95,1.17) |
| Missing |  | 11619 | 2026 | 64 |  | 0.96 (0.75,1.24) |  | 0.95 (0.74,1.22) |
| **p-value^3^** |  |  |  |  |  | 0.26 |  | 0.34 |
| **Marital status** |  |  |  |  |  |  |  |  |
| Single / Widowed / Divorced / Separated |  | 108137 | 19396 | 604 |  | 1.00 |  | 1.00 |
| Married / Living with partner |  | 480952 | 83072 | 2905 |  | 1.07 (0.98,1.17) |  | 1.00 (0.91,1.09) |
| Missing |  | 5193 | 981 | 38 |  | 1.37 (0.99,1.91) |  | 1.39 (1.00,1.93) |
| **p-value^3^** |  |  |  |  |  | 0.13 |  | 0.91 |
| **Comorbidity^4^** |  |  |  |  |  |  |  |  |
| 0 |  | 503559 | 85917 | 3104 |  | 1.00 |  | 1.00 |
| 1 |  | 44840 | 8126 | 235 |  | 0.68 (0.59,0.78) |  | 0.75 (0.66,0.86) |
| 2+ |  | 45882 | 9406 | 208 |  | 0.57 (0.49,0.66) |  | 0.67 (0.58,0.78) |
| **p-value^3^** |  |  |  |  |  | <0.0001 |  | <0.0001 |
| **Frequency PSA testing per 5 years^5^** | | |  |  |  |  |  |  |
| No record for PSA testing or monitoring |  | 62803 | 11767 | 59 |  | 1.00 |  | 1.00 |
| No record for PSA testing + 1 monitoring^6^ |  | 71164 | 12285 | 837 |  | 9.33(7.16, 12.15) ((7.16,12.01) |  | 8.91 (6.83,11.63) (6.83,11.63) |
| <1 |  | 163091 | 27869 | 827 |  | 4.44 (3.41,5.78) |  | 4.29 (3.29,5.59) |
| 1 - <2 |  | 221439 | 38359 | 1103 |  | 4.13 (3.18,5.37) |  | 3.95 (3.03,5.14) |
| 2 - <3 |  | 47962 | 8309 | 540 |  | 8.79 (6.71,11.51) |  | 8.28 (6.31,10.86) |
| 3+ |  | 27823 | 4860 | 181 |  | 4.81 (3.58,6.46) |  | 4.47 (3.32,6.01) |
| **p-value^3^** |  |  |  |  |  | <0.0001 |  | <0.0001 |
| **Frequency GP visits per 5 years^7^** | | |  |  |  |  |  |  |
| 0 - <12 |  | 153128 | 26383 | 593 |  | 1.00 |  | 1.00 |
| 12 - <21 |  | 148831 | 25439 | 966 |  | 1.27 (1.14,1.41) |  | 1.17 (1.05,1.29) |
| 21 - <36 |  | 149978 | 25906 | 1009 |  | 1.10 (0.99,1.23) |  | 1.03 (0.93,1.15) |
| 36- 421 |  | 142346 | 25721 | 979 |  | 1.02 (0.92,1.14) |  | 1.03 (0.92,1.16) |
| **p-value^3^** |  |  |  |  |  | <0.0001 |  | 0.0084 |

^1^Adjusted for Age (as underlying time variable).

^2^ Additionally adjusted for region of birth, health cover, income, qualification, place of residence, marital status, Charlson’s comorbidity index unless variable is the exposure of interest, frequency of PSA testing and frequency of GP visits, family history of cancer, BMI, smoking alcohol, physical activity, lower urinary tract symptoms, vasectomy, erectile dysfunction, medications for BPH and medications for diabetes.

^3^P-values are based on tests that exclude the HRs of missing value categories.

^4^Based on Charlson’s comorbidity index for MBS records before PC diagnosis or censoring date.

^5^MBS records for PSA testing (MBS code 66655) and monitoring (MBS code 66656, 66659, 66660) before PC diagnosis or censoring date.

^6^ Men with no record for PSA testing (MBS code 66655) but have at least one record for monitoring (MBS code 66656, 66659, 66660).

^7^MBS records for GP visits before PC diagnosis or censoring date.

Supplementary Table 3- Joint Cox regression (HR, 95% CI) for prostate cancer diagnosis and personal, behavioural and health-related characteristics, after excluding the first year of follow-up after study entry for male participants after the date of enrolment in the 45 and Up Study (n=106,996).

| Variable |  |  | PC cases (n) | | | |  | HR^1^ (95% CI) | |  | HR^2^ (95% CI) | |
| --- | --- | --- | --- | --- | --- | --- | --- | --- | --- | --- | --- | --- |
|  | | | Localised  N=1956 | | Advanced  N=457 | |  | Localised | Advanced |  | Localised | Advanced |
| **Family History of cancer** | | |  | |  | |  |  |  |  |  |  |
| No cancer |  |  | 1062 | | 279 | |  | 1.00 | 1.00 |  | 1.00 | 1.00 |
| Prostate cancer (PC) only |  |  | 171 | | 36 | |  | 1.57 (1.34, 1.85) | 1.27 (0.90, 1.80) |  | 1.46 (1.24, 1.71) | 1.16 (0.82, 1.65) |
| PC and BC and/or OvC |  |  | 354 | | 76 | |  | 1.31 (1.16, 1.48) | 1.08 (0.84, 1.39) |  | 1.27 (1.12, 1.43) | 1.04 (0.81, 1.34) |
| Other cancers |  |  | 369 | | 84 | |  | 0.99 (0.88, 1.12) | 0.87 (0.68, 1.10) |  | 0.96 (0.85, 1.08) | 0.83 (0.65, 1.06) |
| p-value^3^ |  |  |  | |  | |  | 0.39 | |  | 0.37 | |
| **Family History of prostate cancer^4^** | | |  | |  | |  |  |  |  |  |  |
| None |  |  | 1632 | | 404 | |  | 1.00 | 1.00 |  | 1.00 | 1.00 |
| Father only |  |  | 240 | | 51 | |  | 1.78 (1.55, 2.04) | 1.54 (1.15, 2.06) |  | 1.68 (1.46, 1.92) | 1.44 (1.08, 1.93) |
| Brother only |  |  | 67 | | 15 | |  | 1.65 (1.29, 2.11) | 1.49 (0.89, 2.50) |  | 1.55 (1.21, 1.98) | 1.39 (0.83, 2.33) |
| Father and Brother |  |  | 17 | | 5 | |  | 2.40 (1.49, 3.86) | 2.82 (1.17, 6.82) |  | 2.07 (1.29, 3.35) | 2.59 (1.07, 6.27) |
| p-value^3^ |  |  |  | |  | |  |  | 0.80 |  |  | 0.76 |
| **BMI (kg/m^2^)** |  |  |  | |  | |  |  |  |  |  |  |
| 15-24.9 |  |  | 567 | | 128 | |  | 1.00 | 1.00 |  | 1.00 | 1.00 |
| 25-29.9 |  |  | 916 | | 202 | |  | 1.02 (0.92, 1.13) | 1.03 (0.82, 1.29) |  | 1.01 (0.91, 1.13) | 1.04 (0.83, 1.30) |
| >30 |  |  | 358 | | 104 | |  | 0.87 (0.76, 1.00) | 1.17 (0.90, 1.52) |  | 0.95 (0.82, 1.09) | 1.37 (1.04, 1.80) |
| Missing |  |  | 115 | | 41 | |  | 0.91 (0.75, 1.11) | 1.45 (1.02, 2.06) |  | 0.92 (0.76, 1.13) | 1.51 (1.06, 2.15) |
| p-value^3^ |  |  |  | |  | |  | 0.081  .08 | |  | 0.024 | |
| **Height (cm)^4^** |  |  |  | |  | |  |  |  |  |  |  |
| 55-170.18 (inclusive) |  |  | 497 | | 108 | |  | 1.00 | 1.00 |  | 1.00 | 1.00 |
| 170.18-176.53 |  |  | 488 | | 107 | |  | 1.00 (0.88, 1.13) | 1.04 (0.79, 1.35) |  | 0.97 (0.86, 1.10) | 1.00 (0.76, 1.31) |
| 176.53-180.34 |  |  | 511 | | 126 | |  | 1.00 (0.88, 1.13) | 1.17 (0.91, 1.52) |  | 0.96 (0.85, 1.09) | 1.11 (0.85, 1.44) |
| 180.34-241.30 |  |  | 389 | | 109 | |  | 0.98 (0.85, 1.12) | 1.32 (1.01, 1.73) |  | 0.95 (0.83, 1.09) | 1.26 (0.96, 1.66) |
| Missing |  |  | 71 | | 25 | |  | 0.93 (0.72, 1.19) | 1.52 (0.98, 2.35) |  | 0.90 (0.70, 1.15) | 1.45 (0.94, 2.25) |
| p-value^3^ |  |  |  | |  | |  | 0.19 | |  | 0.26 | |
| **Alcohol consumption (drinks/week)** |  |  |  | |  | |  |  |  |  |  |  |
| Non-drinkers |  |  | 406 | | 109 | |  | 1.05 (0.89, 1.24) | 1.20 (0.86, 1.67) |  | 1.08 (0.91, 1.27) | 1.25 (0.90, 1.75) |
| 1-3 |  |  | 225 | | 52 | |  | 1 | 1 |  | 1.00 | 1.00 |
| 4-14 |  |  | 818 | | 175 | |  | 1.26 (1.08, 1.46) | 1.17 (0.86, 1.59) |  | 1.23 (1.06, 1.43) | 1.12 (0.82, 1.53) |
| 15-28 |  |  | 322 | | 97 | |  | 1.16 (0.98, 1.37) | 1.54 (1.10, 2.16) |  | 1.17 (0.98, 1.39) | 1.49 (1.05, 2.09) |
| 29+ |  |  | 156 | | 36 | |  | 1.19 (0.97, 1.47) | 1.22 (0.80, 1.87) |  | 1.24 (1.01, 1.53) | 1.21 (0.78, 1.86) |
| Missing |  |  | 29 | | 6 | |  | 1.08 (0.73, 1.59) | 0.93 (0.40, 2.16) |  | 1.21 (0.82, 1.79) | 1.02 (0.43, 2.38) |
| p-value^3^ |  |  |  | |  | |  | 0.13 | |  | 0.14 | |
| **Physical activity^3^ (hours/week)** |  |  |  | |  | |  |  |  |  |  |  |
| 0-<4 |  |  | 268 | | 69 | |  | 1.00 | 1.00 |  | 1.00 | 1.00 |
| 4-<7 |  |  | 283 | | 78 | |  | 1.01 (0.86, 1.20) | 1.10 (0.80, 1.53) |  | 0.96 (0.81, 1.14) | 1.10 (0.79, 1.52) |
| 7-<11 |  |  | 480 | | 98 | |  | 1.11 (0.95, 1.28) | 0.89 (0.66, 1.21) |  | 1.04 (0.89, 1.21) | 0.88 (0.64, 1.20) |
| 11-<18 |  |  | 487 | | 111 | |  | 1.10 (0.95, 1.27) | 0.99 (0.74, 1.34) |  | 1.02 (0.88, 1.19) | 0.98 (0.72, 1.32) |
| 18+ |  |  | 414 | | 113 | |  | 1.05 (0.90, 1.23) | 1.15 (0.85, 1.55) |  | 0.99 (0.84, 1.16) | 1.11 (0.82, 1.51) |
| Missing |  |  | 24 | | 6 | |  | 0.93 (0.61, 1.42) | 0.90 (0.39, 2.07) |  | 0.93 (0.61, 1.41) | 0.89 (0.38, 2.05) |
| p-value^3^ |  |  |  | |  | |  | 0.26 | |  | 0.32 | |
| **Smoking** |  |  |  | |  | |  |  |  |  |  |  |
| Never |  |  | 1009 | | 244 | |  | 1.00 | 1.00 |  | 1.00 | 1.00 |
| Ever |  |  | 947 | | 231 | |  | 0.85 (0.78, 0.93) | 0.86 (0.72, 1.03) |  | 0.88 (0.80, 0.96) | 0.89 (0.74, 1.08) |
| Missing |  |  |  | |  | |  |  | NA |  |  | NA |
| p-value^3^ |  |  |  | |  | |  | 0.89 | |  | 0.87 | |
| **LUTS** |  |  |  | |  | |  |  |  |  |  |  |
| Mild (0-5) |  |  | 1226 | | | 306 |  | 1.00 | 1.00 |  | 1.00 | 1.00 |
| Moderate (6-11) |  |  | 387 | | | 92 |  | 1.34 (1.19, 1.50) | 1.25 (0.99, 1.58) |  | 1.30 (1.15, 1.46) | 1.28 (1.01, 1.63) |
| Severe (12-21) |  |  | 88 | | | 19 |  | 1.65 (1.33, 2.06) | 1.40 (0.88, 2.23) |  | 1.67 (1.34, 2.08) | 1.54 (0.96, 2.48) |
| Missing |  |  | 255 | | | 58 |  | 1.04 (0.91, 1.19) | 0.89 (0.67, 1.19) |  | 1.06 (0.92, 1.21) | 0.92 (0.69, 1.23) |
| p-value^3^ |  |  |  | |  | |  | 0.75 | |  | 0.96 | |
| **Erectile dysfunction** |  |  |  | |  | |  |  |  |  |  |  |
| No |  |  | 1069 | | 271 | | <.0001 | 1.00 | 1.00 |  | 1.00 | 1.00 |
| Yes |  |  | 664 | | 156 | |  | 1.00 (0.90, 1.12) | 0.85 (0.69, 1.06) |  | 1.06 (0.95, 1.19) | 0.92 (0.74, 1.16) |
| Missing |  |  | 223 | | 48 | |  | 0.88 (0.76, 1.03) | 0.68 (0.49, 0.94) |  | 0.97 (0.83, 1.13) | 0.74 (0.53, 1.03) |
| p-value^3^ |  |  |  | |  | |  | 0.19 | |  | 0.28 | |
| **Vasectomy** |  |  |  | |  | |  |  |  |  |  |  |
| No |  |  | 1453 | | 337 | |  | 1.00 | 1.00 |  | 1.00 | 1.00 |
| Yes |  |  | 503 | | 138 | |  | 1.09 (0.98, 1.21) | 1.34 (1.10, 1.64) |  | 1.02 (0.92, 1.14) | 1.28 (1.04, 1.57) |
| p-value^3^ |  |  |  | |  | |  | 0.069 | |  | 0.056 | |
| **Prescription for BPH^5^** |  |  |  | |  | |  |  |  |  |  |  |
| No |  |  | 1766 | | 439 | |  | 1.00 | 1.00 |  | 1.00 | 1.00 |
| Yes |  |  | 190 | | 36 | |  | 0.92 (0.79, 1.07) | 0.69 (0.49, 0.97) |  | 0.82 (0.70, 0.96) | 0.65 (0.46, 0.93) |
| p-value^3^ |  |  |  | |  | |  | 0.13 | |  | 0.25 | |
| **Prescription for diabetes^5^** | | | |  | | |  |  |  |  |  |  |
| None |  |  | 1598 | | 398 | |  | 1.00 | 1.00 |  | 1.00 | 1.00 |
| Only metformin |  |  | 62 | | 15 | |  | 0.52 (0.40, 0.67) | 0.51 (0.30, 0.86) |  | 0.57 (0.44, 0.74) | 0.56 (0.33, 0.94) |
| Only Non-Metformin diabetes medication |  |  | 25 | | 7 | |  | 0.61 (0.41, 0.91) | 0.67 (0.32, 1.42) |  | 0.72 (0.48, 1.07) | 0.78 (0.37, 1.67) |
| Mixture of metformin and non-metformin |  |  | 271 | | 55 | |  | 0.73 (0.64, 0.83) | 0.59 (0.45, 0.79) |  | 0.80 (0.69, 0.91) | 0.65 (0.48, 0.87) |
| p-value^3^ |  |  |  | |  | |  | 0.63 | |  | 0.64 | |

^1^Adjusted for Age as the underlying time variable. .

^2^Adjusted for Age, region of birth, health cover, income, qualification, place of residence, marital status, Charlson’s comorbidity index, frequency of PSA testing, frequency of primary health care visits, family history of cancer, BMI, smoking alcohol, physical activity, lower urinary tract symptoms, vasectomy, erectile dysfunction, medications for BPH and medications for diabetes, unless variable is the exposure of interest.

^3^ P-values are for tests of HR equality between PC stage excluding the HRs of missing value categories.

^4^Family history of prostate cancer was not adjusted for family history of cancer; height was not adjusted for BMI;

^5^ Information obtained from PBS records before PC diagnosis or censoring date.

Supplementary Table 4 Hazard ratios (HR) and 95% CI for diagnosis of prostate cancer and sociodemographic and health-related characteristics, after excluding high PSA testers in the 45 and Up Study (n=102,542).

| **Characteristics** | Main model | | | | | | | | Excluding High testers | | | | | | | | |  |
| --- | --- | --- | --- | --- | --- | --- | --- | --- | --- | --- | --- | --- | --- | --- | --- | --- | --- | --- |
|  | HR^1^ (95% CI) | | | | HR^2^ (95% CI) | | | | HR^1^ (95% CI) | | | | HR^2^ (95% CI) | | | | |  |
| **Comorbidity^4^** |  |  |  | p-value^3^ |  |  |  | p-value^3^ |  |  |  | p-value^3^ | |  |  |  | p-value^3^ | |
| 0 | 1 |  |  | <0.0001 | 1.00 |  |  | <0.0001 | 1 |  |  | <0.0001 | | 1.00 |  |  | <0.0001 | |
| 1 | 0.63 | 0.55 | 0.71 |  | 0.70 | 0.61 | 0.79 |  | 0.62 | 0.54 | 0.70 |  | | 0.68 | 0.60 | 0.78 |  | |
| 2+ | 0.57 | 0.50 | 0.64 |  | 0.67 | 0.59 | 0.77 |  | 0.58 | 0.51 | 0.66 |  | | 0.68 | 0.59 | 0.78 |  | |
| **Frequency PSA testing per 5 years^5^** |  |  |  |  |  |  |  |  |  |  |  |  | |  |  |  |  | |
| No record for PSA testing or monitoring | 1 |  |  | <0.0001 | 1.00 |  |  | <0.0001 | 1 |  |  | <0.0001 | | 1.00 |  |  | <0.0001 | |
| No record for PSA testing + 1 monitoring | 7.31 | 5.93 | 9.03 |  | 6.99 | 5.66 | 8.64 |  | 7.29 | 5.91 | 9.00 |  | | 6.99 | 5.66 | 8.64 |  | |
| <1 | 2.78 | 2.25 | 3.44 |  | 2.70 | 2.18 | 3.34 |  | 2.77 | 2.24 | 3.43 |  | | 2.69 | 2.18 | 3.33 |  | |
| 1 - <2 | 3.16 | 2.56 | 3.89 |  | 3.02 | 2.45 | 3.73 |  | 3.14 | 2.55 | 3.87 |  | | 3.01 | 2.44 | 3.72 |  | |
| 2 - <3 | 6.18 | 4.98 | 7.68 |  | 5.85 | 4.70 | 7.28 |  | 6.14 | 4.94 | 7.63 |  | | 5.82 | 4.68 | 7.24 |  | |
| 3+ | 5.00 | 3.97 | 6.31 |  | 4.66 | 3.69 | 5.89 |  |  |  |  |  | |  |  |  |  | |
| **Frequency GP visits per 5 years^6^** |  |  |  |  |  |  |  |  |  |  |  |  | |  |  |  |  | |
| 0 - 14.4 | 1 |  |  | <0.0001 | 1.00 |  |  | 0.016 | 1 |  |  | <0.0001 | | 1.00 |  |  | 0.0084 | |
| 14.4< - 25.21 | 1.25 | 1.14 | 1.38 |  | 1.14 | 1.03 | 1.25 |  | 1.25 | 1.13 | 1.38 |  | | 1.14 | 1.04 | 1.26 |  | |
| 25.21< - 41.95 | 1.11 | 1.00 | 1.22 |  | 1.02 | 0.93 | 1.13 |  | 1.10 | 0.99 | 1.21 |  | | 1.02 | 0.92 | 1.13 |  | |
| 41.95< - 523.98 | 1.02 | 0.92 | 1.13 |  | 1.02 | 0.92 | 1.14 |  | 0.99 | 0.89 | 1.10 |  | | 1.00 | 0.89 | 1.12 |  | |
| **Region of birth** |  |  |  |  |  |  |  |  |  |  |  |  | |  |  |  |  | |
| Australia or New Zealand | 1 |  |  | <0.0001 | 1.00 |  |  | <0.0001 | 1 |  |  | <0.0001 | | 1.00 |  |  | <0.0001 | |
| Other countries | 0.80 | 0.74 | 0.86 |  | 0.84 | 0.78 | 0.91 |  | 0.79 | 0.74 | 0.86 |  | | 0.84 | 0.77 | 0.90 |  | |
| **Health cover** |  |  |  |  |  |  |  |  |  |  |  |  | |  |  |  |  | |
| None | 1 |  |  | 0.0006 | 1.00 |  |  | 0.20 | 1 |  |  | 0.0005 | | 1.00 |  |  | 0.24 | |
| Health care concession card | 1.11 | 1.00 | 1.23 |  | 1.07 | 0.96 | 1.19 |  | 1.08 | 0.97 | 1.20 |  | | 1.05 | 0.95 | 1.17 |  | |
| Private health insurance | 1.19 | 1.09 | 1.31 |  | 1.10 | 0.99 | 1.21 |  | 1.19 | 1.08 | 1.31 |  | | 1.09 | 0.98 | 1.21 |  | |
| Missing | 1.08 | 0.86 | 1.36 |  | 1.12 | 0.89 | 1.41 |  | 1.05 | 0.83 | 1.32 |  | | 1.09 | 0.86 | 1.38 |  | |
| **Income** |  |  |  |  |  |  |  |  |  |  |  |  | |  |  |  |  | |
| Less than $19,999 per year | 1 |  |  | 0.93 | 1.00 |  |  | **0.028** | 1 |  |  | 0.82 | | 1.00 |  |  | **0.23** | |
| $20,000-$29,999 per year | 1.00 | 0.90 | 1.12 |  | 0.92 | 0.82 | 1.03 |  | 1.01 | 0.90 | 1.13 |  | | 0.93 | 0.83 | 1.04 |  | |
| $30,000-$39,999 per year | 0.97 | 0.86 | 1.10 |  | 0.85 | 0.75 | 0.96 |  | 1.00 | 0.88 | 1.13 |  | | 0.87 | 0.77 | 0.99 |  | |
| $40,000-$49,999 per year | 0.99 | 0.87 | 1.12 |  | 0.85 | 0.74 | 0.97 |  | 1.02 | 0.90 | 1.16 |  | | 0.88 | 0.77 | 1.01 |  | |
| $50,000-$69,999 per year | 1.05 | 0.94 | 1.17 |  | 0.88 | 0.78 | 0.99 |  | 1.08 | 0.96 | 1.22 |  | | 0.91 | 0.80 | 1.03 |  | |
| $70,000 or more per year | 1.01 | 0.91 | 1.11 |  | 0.83 | 0.74 | 0.94 |  | 1.05 | 0.95 | 1.16 |  | | 0.87 | 0.77 | 0.99 |  | |
| Missing | 0.91 | 0.83 | 1.01 |  | 0.87 | 0.78 | 0.96 |  | 0.92 | 0.83 | 1.02 |  | | 0.87 | 0.78 | 0.97 |  | |
| **Qualification** |  |  |  |  |  |  |  |  |  |  |  |  | |  |  |  |  | |
| No school certificate | 1 |  |  | 0.60 | 1.00 |  |  | 0.81 | 1 |  |  | 0.84 | | 1.00 |  |  | 0.78 | |
| School or intermediate certificate | 1.10 | 0.98 | 1.23 |  | 1.04 | 0.92 | 1.17 |  | 1.08 | 0.96 | 1.22 |  | | 1.02 | 0.90 | 1.15 |  | |
| Higher school or leaving certificate | 1.06 | 0.93 | 1.21 |  | 1.05 | 0.92 | 1.21 |  | 1.07 | 0.93 | 1.23 |  | | 1.05 | 0.91 | 1.20 |  | |
| Trade or apprenticeship | 1.04 | 0.93 | 1.16 |  | 0.99 | 0.88 | 1.11 |  | 1.05 | 0.93 | 1.18 |  | | 0.99 | 0.88 | 1.11 |  | |
| Certificate or diploma | 1.10 | 0.98 | 1.23 |  | 1.03 | 0.92 | 1.16 |  | 1.07 | 0.95 | 1.21 |  | | 1.00 | 0.88 | 1.12 |  | |
| University degree or higher | 1.06 | 0.95 | 1.18 |  | 0.99 | 0.88 | 1.12 |  | 1.04 | 0.93 | 1.17 |  | | 0.95 | 0.84 | 1.08 |  | |
| Missing | 1.06 | 0.83 | 1.34 |  | 1.12 | 0.88 | 1.43 |  | 1.05 | 0.82 | 1.34 |  | | 1.10 | 0.86 | 1.41 |  | |
| **Place of residence** |  |  |  |  |  |  |  |  |  |  |  |  | |  |  |  |  | |
| Major Cities | 1 |  |  | 0.27 | 1.00 |  |  | 0.39 | 1 |  |  | 0.33 | | 1.00 |  |  | 0.43 | |
| Inner Regional | 1.05 | 0.98 | 1.12 |  | 1.04 | 0.97 | 1.11 |  | 1.05 | 0.98 | 1.13 |  | | 1.04 | 0.98 | 1.12 |  | |
| Outer Regional/Remote | 1.06 | 0.96 | 1.17 |  | 1.05 | 0.95 | 1.16 |  | 1.04 | 0.94 | 1.16 |  | | 1.04 | 0.94 | 1.15 |  | |
| Missing | 0.95 | 0.76 | 1.20 |  | 0.95 | 0.75 | 1.19 |  | 0.94 | 0.74 | 1.19 |  | | 0.93 | 0.74 | 1.18 |  | |
| **Marital status** |  |  |  |  |  |  |  |  |  |  |  |  | |  |  |  |  | |
| Single/Widowed / Divorced Separated | 1 |  |  | 0.086 | 1.00 |  |  | 0.98 | 1 |  |  | 0.11 | | 1.00 |  |  | 0.89 | |
| Married/Living with partner | 1.07 | 0.99 | 1.16 |  | 1.00 | 0.92 | 1.09 |  | 1.07 | 0.99 | 1.16 |  | | 0.99 | 0.91 | 1.08 |  | |
| Missing | 1.27 | 0.93 | 1.73 |  | 1.28 | 0.94 | 1.75 |  | 1.30 | 0.95 | 1.79 |  | | 1.31 | 0.96 | 1.80 |  | |

^1^Adjusted for Age (as underlying time variable)

^2^ Additionally adjusted for region of birth, health cover, income, qualification, place of residence, marital status, Charlson’s comorbidity index unless variable is the exposure of interest, frequency of PSA testing and frequency of primary health care visits, family history of cancer, BMI, smoking alcohol, physical activity, lower urinary tract symptoms, vasectomy, erectile dysfunction, medications for BPH and medications for diabetes.

^3^P-values are based on tests that exclude the HRs of missing value categories.

^4^Based on Charlson’s comorbidity index for MBS records before PC diagnosis or censoring date.

^5^MBS records for PSA testing (MBS code 66655) and monitoring (MBS code 66656, 66659, 66660) before PC diagnosis or censoring date.

^6^ MBS records for GP visits before PC diagnosis or censoring date.

Supplementary Table5 Joint Cox regression (HR, 95% CI) for prostate cancer diagnosis and personal, behavioural and health-related factors, excluding high PSA testers in the 45 and Up Study (n=102,542).

| **Variable** | **Person years** | **PC cases (n)** | |  | HR^1^ (95% CI) | |  | HR^2^ (95% CI) | |
| --- | --- | --- | --- | --- | --- | --- | --- | --- | --- |
|  |  | **Localised n=2197** | **Advanced n=511** |  | **Localised** | **Advanced** |  | **Localised** | **Advanced** |
| **Family History of cancer^3^** |  |  |  |  |  |  |  |  |  |
| No cancer | 332354 | 1198 | 299 |  | 1.00 | 1.00 |  | 1.00 | 1.00 |
| Prostate cancer (PC) only | 34189 | 203 | 39 |  | 1.68 (1.45, 1.95) | 1.30 (0.93, 1.81) |  | 1.54 (1.33, 1.79) | 1.18 (0.85, 1.65) |
| PC and BC or OvC^4^ | 83653 | 392 | 82 |  | 1.29 (1.15, 1.44) | 1.09 (0.85, 1.39) |  | 1.25 (1.11, 1.40) | 1.05 (0.82, 1.34) |
| Other cancers | 116573 | 404 | 91 |  | 0.96 (0.86, 1.07) | 0.87 (0.69, 1.10) |  | 0.93 (0.83, 1.04) | 0.84 (0.67, 1.07) |
| p-value^5^ |  |  |  |  | 0.39 | |  | 0.36 | |
| **Family History of prostate cancer^6^** |  |  |  |  |  |  |  |  |  |
| None | 509276 | 1828 | 435 |  | 1.00 | 1.00 |  | 1.00 | 1.00 |
| Father only | 44881 | 274 | 54 |  | 1.85 (1.63, 2.10) | 1.54 (1.16, 2.04) |  | 1.75 (1.54, 1.98) | 1.43 (1.08, 1.91) |
| Brother only | 10652 | 76 | 17 |  | 1.66 (1.32, 2.09) | 1.57 (0.97, 2.55) |  | 1.55 (1.23, 1.95) | 1.45 (0.89, 2.37) |
| Father and Brother | 1960 | 19 | 5 |  | 2.42 (1.54, 3.80) | 2.66 (1.10, 6.42) |  | 2.05 (1.31, 3.23) | 2.42 (1.00, 5.85) |
| p-value^5^ |  |  |  |  | 0.69 | |  | 0.64 | |
| **Height (cm)^6^** |  |  |  |  |  |  |  |  |  |
| 55-170.18 (inclusive) | 136582 | 550 | 113 |  | 1.00 | 1.00 |  | 1.00 | 1.00 |
| 170.18-176.53 | 136272 | 552 | 114 |  | 1.03 (0.92, 1.16) | 1.07 (0.82, 1.38) |  | 1.00 (0.89, 1.13) | 1.02 (0.79, 1.33) |
| 176.53-180.34 | 149361 | 573 | 135 |  | 1.02 (0.91, 1.15) | 1.22 (0.95, 1.56) |  | 0.99 (0.88, 1.11) | 1.14 (0.89, 1.47) |
| 180.34-241.30 | 122726 | 436 | 122 |  | 1.01 (0.89, 1.14) | 1.44 (1.11, 1.87) |  | 0.99 (0.87, 1.13) | 1.37 (1.05, 1.78) |
| Missing | 21828 | 86 | 27 |  | 1.01 (0.80, 1.27) | 1.56 (1.02, 2.37) |  | 0.98 (0.78, 1.23) | 1.51 (0.99, 2.30) |
| p-value^5^ |  |  |  |  | 0.063 | |  | 0.11 | |
| **BMI (kg/m^2^)^3^** |  |  |  |  |  |  |  |  |  |
| 15-24.9 | 163233 | 619 | 138 |  | 1.00 | 1.00 |  | 1.00 | 1.00 |
| 25-29.9 | 251792 | 1041 | 223 |  | 1.08 (0.97, 1.19) | 1.07 (0.86, 1.32) |  | 1.07 (0.96, 1.18) | 1.07 (0.87, 1.33) |
| >30 | 116548 | 400 | 105 |  | 0.90 (0.80, 1.02) | 1.11 (0.86, 1.43) |  | 0.98 (0.86, 1.12) | 1.31 (1.00, 1.71) |
| Missing | 35196 | 137 | 45 |  | 0.99 (0.82, 1.19) | 1.47 (1.05, 2.05) |  | 1.00 (0.83, 1.20) | 1.54 (1.10, 2.17) |
| p-value^5^ |  |  |  |  | 0.25 | |  | 0.08 | |
| **Smoking^3^** |  |  |  |  |  |  |  |  |  |
| Never | 279311 | 1112 | 266 |  | 1.00 | 1.00 |  | 1.00 | 1.00 |
| Ever | 287221 | 1085 | 245 |  | 0.87 (0.80, 0.95) | 0.83 (0.70, 0.99) |  | 0.90 (0.83, 0.99) | 0.87 (0.73, 1.04) |
| Missing | 238 |  |  |  |  |  |  |  |  |
| p-value^5^ |  |  |  |  | 0.63 | |  | 0.72 | |
| **Alcohol consumption (drinks/week)^3^** |  |  | |  |  |  |  |  |  |
| Non-drinkers | 129142 | 470 | 120 |  | 1.02 (0.87, 1.18) | 1.25 (0.91, 1.72) |  | 1.05 (0.90, 1.22) | 1.33 (0.96, 1.83) |
| 1-3 | 78048 | 264 | 54 |  | 1.00 | 1.00 |  | 1.00 | 1.00 |
| 4-14 | 216539 | 884 | 188 |  | 1.16 (1.01, 1.33) | 1.21 (0.89, 1.63) |  | 1.14 (0.99, 1.31) | 1.16 (0.86, 1.58) |
| 15-28 | 90753 | 375 | 106 |  | 1.16 (0.99, 1.36) | 1.64 (1.18, 2.28) |  | 1.17 (0.99, 1.37) | 1.58 (1.14, 2.21) |
| 29+ | 43403 | 169 | 38 |  | 1.12 (0.92, 1.35) | 1.26 (0.83, 1.91) |  | 1.16 (0.95, 1.41) | 1.26 (0.83, 1.93) |
| Missing | 8885 | 35 | 5 |  | 1.07 (0.76, 1.53) | 0.72 (0.29, 1.81) |  | 1.23 (0.86, 1.75) | 0.82 (0.33, 2.07) |
| p-value^5^ |  |  |  |  | 0.18 | |  | 0.18 | |
| **Physical activity (hours/week)^3^** |  |  |  |  |  |  |  |  |  |
| 0-<4 | 86348 | 316 | 75 |  | 1.00 | 1.00 |  | 1.00 | 1.00 |
| 4-<7 | 86767 | 320 | 87 |  | 0.99 (0.85, 1.16) | 1.16 (0.85, 1.58) |  | 0.94 (0.81, 1.10) | 1.13 (0.83, 1.54) |
| 7-<11 | 130763 | 535 | 111 |  | 1.07 (0.93, 1.23) | 0.95 (0.71, 1.28) |  | 1.00 (0.87, 1.16) | 0.91 (0.68, 1.23) |
| 11-<18 | 133469 | 548 | 115 |  | 1.07 (0.93, 1.23) | 0.97 (0.73, 1.30) |  | 1.00 (0.87, 1.15) | 0.93 (0.69, 1.25) |
| 18+ | 121866 | 450 | 118 |  | 0.99 (0.86, 1.15) | 1.13 (0.85, 1.51) |  | 0.93 (0.81, 1.08) | 1.07 (0.79, 1.43) |
| Missing | 7557 | 28 | 5 |  | 0.91 (0.62, 1.34) | 0.68 (0.28, 1.68) |  | 0.90 (0.61, 1.33) | 0.67 (0.27, 1.67) |
| p-value^5^ |  |  |  |  | 0.26 | |  | 0.28 | |
| **Self reported LUTS** |  |  |  |  |  |  |  |  |  |
| Mild (0-5) | 397225 | 1342 | 331 |  | 1.00 | 1.00 |  | 1.00 | 1.00 |
| Moderate (6-11) | 83925 | 446 | 95 |  | 1.36 (1.22, 1.52) | 1.16 (0.92, 1.46) |  | 1.33 (1.19, 1.48) | 1.20 (0.95, 1.51) |
| Severe (12-21) | 14772 | 112 | 21 |  | 1.83 (1.50, 2.22) | 1.37 (0.88, 2.13) |  | 1.86 (1.52, 2.26) | 1.54 (0.98, 2.42) |
| Missing | 70849 | 297 | 64 |  | 1.06 (0.94, 1.21) | 0.88 (0.67, 1.15) |  | 1.08 (0.95, 1.23) | 0.92 (0.70, 1.22) |
| p-value^5^ |  |  |  |  | 0.27 | |  | 0.61 | |
| **Prescription for BPH^3,7^** |  |  |  |  |  |  |  |  |  |
| No | 518290 | 1987 | 473 |  | 1.00 | 1.00 |  | 1.00 | 1.00 |
| Yes | 48480 | 210 | 38 |  | 0.87 (0.75, 1.00) | 0.66 (0.47, 0.92) |  | 0.76 (0.65, 0.88) | 0.64 (0.46, 0.90) |
| p-value^5^ |  |  |  |  | 0.13 | |  | 0.38 | |
| **Vasectomy^3^** |  |  |  |  |  |  |  |  |  |
| No | 422413 | 1642 | 362 |  | 1.00 | 1.00 |  | 1.00 | 1.00 |
| Yes | 144357 | 555 | 149 |  | 1.09 (0.99, 1.20) | 1.38 (1.13, 1.67) |  | 1.02 (0.93, 1.13) | 1.30 (1.07, 1.58) |
| p-value^5^ |  |  |  |  | 0.034 | |  | 0.032 | |
| **Erectile Dysfunction^3^** |  |  |  |  |  |  |  |  |  |
| No | 336829 | 1176 | 288 |  | 1.00 | 1.00 |  | 1.00 | 1.00 |
| Yes | 163299 | 776 | 172 |  | 1.01 (0.92, 1.12) | 0.85 (0.69, 1.05) |  | 1.06 (0.96, 1.18) | 0.94 (0.76, 1.17) |
| Missing | 66641 | 245 | 51 |  | 0.84 (0.73, 0.96) | 0.65 (0.48, 0.88) |  | 0.92 (0.80, 1.07) | 0.73 (0.53, 1.01) |
| p-value^5^ |  |  |  |  | 0.13 | |  | 0.31 | |
| **Diabetic medication^3,7^** |  |  |  |  |  |  |  |  |  |
| None | 441582 | 1786 | 429 |  | 1.00 | 1.00 |  | 1.00 | 1.00 |
| Only metformin | 27572 | 73 | 16 |  | 0.54 (0.43, 0.69) | 0.50 (0.31, 0.83) |  | 0.60 (0.47, 0.76) | 0.57 (0.34, 0.95) |
| Only Non-Metformin diabetes medication | 10514 | 31 | 10 |  | 0.66 (0.46, 0.94) | 0.86 (0.46, 1.61) |  | 0.77 (0.54, 1.11) | 1.06 (0.56, 2.01) |
| Mixture of metformin and non-metformin | 87102 | 307 | 56 |  | 0.72 (0.64, 0.81) | 0.55 (0.42, 0.73) |  | 0.79 (0.70, 0.90) | 0.63 (0.47, 0.84) |
| p-value^5^ |  |  |  |  | 0.30 | |  | 0.39 | |

^1^Adjusted for Age as the underlying time variable.

^2^Adjusted for Age, region of birth, health cover, income, qualification, place of residence, marital status, Charlson’s comorbidity index, frequency of PSA testing, frequency of primary health care visits, family history of cancer, BMI, smoking alcohol, physical activity, lower urinary tract symptoms, vasectomy, erectile dysfunction, medications for BPH and medications for diabetes, unless variable is the exposure of interest.

^3^variables used in the fully adjusted model.

^4^ P-values are for tests of HR equality between PC stage excluding the HRs of missing value categories.

^5^Family history of prostate cancer was not adjusted for family history of cancer; height was not adjusted for BMI;

^6^ Information obtained from PBS records before PC diagnosis or censoring date.

Supplementary Table 6- Joint Cox regression (HR,95% CI) for prostate cancer diagnosis and personal, behavioural and health-related factors for 45 and Up Study male participants (n=107,706).

| **Variable** |  | **PC cases (n)** | | |  | **HR^1^ (95% CI)** | | |
| --- | --- | --- | --- | --- | --- | --- | --- | --- |
|  |  | Localised | Non-localised | Unknown |  | Localised | Non-localised | Unknown |
| **Family History of cancer^2^** |  |  |  |  |  |  |  |  |
| No cancer |  | 1287 | 316 | 762 |  | 1.00 | 1.00 | 1.00 |
| Prostate cancer (PC) only |  | 216 | 42 | 91 |  | 1.52 (1.31, 1.75) | 1.20 (0.87, 1.66) | 1.14 (0.92, 1.42) |
| PC and BC and/or OvC^3^ |  | 433 | 90 | 226 |  | 1.28 (1.15, 1.43) | 1.09 (0.86, 1.38) | 1.16 (1.00, 1.34) |
| Other cancers |  | 432 | 95 | 267 |  | 0.93 (0.83, 1.04) | 0.84 (0.66, 1.05) | 0.99 (0.86, 1.14) |
| p-value^4^ |  |  |  |  |  |  | 0.19 |  |
| **Family History of prostate cancer^5^** |  |  |  |  |  |  |  |  |
| None |  | 1970 | 462 | 1168 |  | 1.00 | 1.00 | 1.00 |
| Father only |  | 292 | 57 | 111 |  | 1.71 (1.51, 1.93) | 1.42 (1.07, 1.87) | 1.21 (0.99, 1.47) |
| Brother only |  | 84 | 19 | 53 |  | 1.57 (1.26, 1.95) | 1.51 (0.95, 2.40) | 1.48 (1.12, 1.95) |
| Father and Brother |  | 22 | 5 | 14 |  | 2.14 (1.41, 3.26) | 2.18 (0.90, 5.27) | 2.29 (1.35, 3.88) |
| p-value^4^ |  |  |  |  |  |  | 0.17 |  |
| **BMI (kg/m^2^)^2^** |  |  |  |  |  |  |  |  |
| 15-24.9 |  | 669 | 149 | 412 |  | 1.00 | 1.00 | 1.00 |
| 25-29.9 |  | 1121 | 233 | 614 |  | 1.06 (0.96, 1.17) | 1.04 (0.84, 1.28) | 1.03 (0.90, 1.16) |
| >30 |  | 431 | 113 | 239 |  | 0.98 (0.87, 1.12) | 1.31 (1.01, 1.69) | 1.04 (0.88, 1.23) |
| Missing |  | 147 | 48 | 81 |  | 1.00 (0.84, 1.20) | 1.54 (1.11, 2.14) | 0.91 (0.72, 1.16) |
| p-value^4^ |  |  |  |  |  |  | 0.19 |  |
| **Height (cm)^5^** |  |  |  |  |  |  |  |  |
| 55-170.18 (inclusive) |  | 593 | 119 | 343 |  | 1.00 | 1.00 | 1.00 |
| 170.18-176.53 |  | 592 | 122 | 349 |  | 0.99 (0.89, 1.11) | 1.04 (0.80, 1.34) | 1.09 (0.94, 1.27) |
| 176.53-180.34 |  | 625 | 145 | 341 |  | 1.00 (0.89, 1.12) | 1.17 (0.92, 1.50) | 1.07 (0.92, 1.25) |
| 180.34-241.30 |  | 466 | 127 | 259 |  | 0.98 (0.86, 1.11) | 1.36 (1.05, 1.76) | 1.13 (0.96, 1.33) |
| Missing |  | 92 | 30 | 54 |  | 0.98 (0.79, 1.22) | 1.61 (1.08, 2.41) | 1.05 (0.79, 1.40) |
| p-value^4^ |  |  |  |  |  |  | 0.30 |  |
| **Alcohol consumption^2^ (drinks/week)** |  |  |  |  |  |  |  |  |
| Non-drinkers |  | 500 | 125 | 318 |  | 1.06 (0.92, 1.23) | 1.27 (0.93, 1.73) | 0.95 (0.79, 1.14) |
| 1-3 |  | 280 | 59 | 184 |  | 1.00 | 1.00 | 1.00 |
| 4-14 |  | 967 | 204 | 520 |  | 1.17 (1.03, 1.34) | 1.15 (0.86, 1.54) | 0.94 (0.80, 1.12) |
| 15-28 |  | 404 | 108 | 211 |  | 1.17 (1.00, 1.37) | 1.46 (1.06, 2.02) | 0.93 (0.76, 1.14) |
| 29+ |  | 181 | 41 | 94 |  | 1.16 (0.96, 1.40) | 1.23 (0.82, 1.85) | 0.94 (0.73, 1.21) |
| Missing |  | 36 | 6 | 19 |  | 1.21 (0.85, 1.72) | 0.91 (0.39, 2.11) | 0.85 (0.53, 1.37) |
| p-value^4^ |  |  |  |  |  |  | 0.24 |  |
| **Physical activity^2^ (hours/week)** |  |  |  |  |  |  |  |  |
| 0-<4 |  | 335 | 78 | 184 |  | 1.00 | 1.00 | 1.00 |
| 4-<7 |  | 339 | 91 | 196 |  | 0.93 (0.80, 1.08) | 1.12 (0.83, 1.52) | 1.02 (0.83, 1.24) |
| 7-<11 |  | 585 | 118 | 347 |  | 1.01 (0.89, 1.16) | 0.92 (0.69, 1.23) | 1.12 (0.93, 1.34) |
| 11-<18 |  | 592 | 125 | 334 |  | 1.00 (0.87, 1.14) | 0.96 (0.72, 1.27) | 1.07 (0.89, 1.29) |
| 18+ |  | 488 | 125 | 274 |  | 0.94 (0.82, 1.09) | 1.08 (0.81, 1.44) | 1.03 (0.85, 1.25) |
| Missing |  | 29 | 6 | 11 |  | 0.88 (0.60, 1.29) | 0.77 (0.33, 1.76) | 0.56 (0.30, 1.02) |
| p-value^4^ |  |  |  |  |  |  | 0.62 |  |
| **Smoking^2^** |  |  |  |  |  |  |  |  |
| Never |  | 1204 | 281 | 671 |  | 1.00 | 1.00 | 1.00 |
| Ever |  | 1164 | 262 | 675 |  | 0.90 (0.83, 0.98) | 0.89 (0.74, 1.06) | 0.94 (0.84, 1.05) |
| Missing |  |  |  |  |  |  | NA |  |
| p-value^4^ |  |  |  |  |  |  | 0.84 |  |
| **LUTS^2^** |  |  |  |  |  |  |  |  |
| Mild (0-5) |  | 1438 | 352 | 769 |  | 1.00 | 1.00 | 1.00 |
| Moderate (6-11) |  | 490 | 103 | 276 |  | 1.38 (1.24, 1.53) | 1.23 (0.98, 1.54) | 1.31 (1.14, 1.51) |
| Severe (12-21) |  | 122 | 21 | 63 |  | 1.93 (1.60, 2.34) | 1.46 (0.93, 2.29) | 1.63 (1.25, 2.12) |
| Missing |  | 318 | 67 | 238 |  | 1.09 (0.96, 1.24) | 0.90 (0.69, 1.18) | 1.25 (1.07, 1.45) |
| p-value^4^ |  |  |  |  |  |  | 0.65 |  |
| **Erectile dysfunction^2^** |  |  |  |  |  |  |  |  |
| No |  | 1256 | 305 | 618 | <.0001 | 1.00 | 1.00 | 1.00 |
| Yes |  | 848 | 183 | 553 |  | 1.10 (1.00, 1.22) | 0.94 (0.76, 1.16) | 1.07 (0.94, 1.22) |
| Missing |  | 264 | 55 | 175 |  | 0.95 (0.82, 1.10) | 0.74 (0.54, 1.01) | 0.95 (0.79, 1.14) |
| p-value^4^ |  |  |  |  |  |  | 0.24 |  |
| **Vasectomy^2^** |  |  |  |  |  |  |  |  |
| No |  | 1759 | 386 | 1036 |  | 1.00 | 1.00 | 1.00 |
| Yes |  | 609 | 157 | 310 |  | 1.04 (0.95, 1.14) | 1.28 (1.06, 1.55) | 1.07 (0.94, 1.21) |
| p-value^3^ |  |  |  |  |  |  | 0.15 |  |
| **Prescription for BPH^2,6^** |  |  |  |  |  |  |  |  |
| No |  | 2143 | 503 | 1188 |  | 1.00 | 1.00 | 1.00 |
| Yes |  | 225 | 40 | 158 |  | 0.76 (0.66, 0.87) | 0.63 (0.45, 0.89) | 0.81 (0.69, 0.97) |
| p-value^4^ |  |  |  |  |  |  | 0.41 |  |
| **Prescription for diabetes^2,6^** |  |  |  |  |  |  |  |  |
| None |  | 1945 | 458 | 1102 |  | 1.00 | 1.00 | 1.00 |
| Only metformin |  | 73 | 17 | 48 |  | 0.55 (0.43, 0.69) | 0.56 (0.34, 0.92) | 0.61 (0.45, 0.81) |
| Only Non-Metformin diabetes medication |  | 32 | 10 | 24 |  | 0.75 (0.52, 1.06) | 0.99 (0.52, 1.88) | 0.89 (0.59, 1.34) |
| Mixture of metformin and non-metformin |  | 318 | 58 | 172 |  | 0.76 (0.67, 0.86) | 0.61 (0.45, 0.81) | 0.68 (0.57, 0.80) |
| p-value^4^ |  |  |  |  |  |  | 0.68 |  |

^1^Adjusted for Age, region of birth, health cover, income, qualification, place of residence, marital status, Charlson’s comorbidity index, frequency of PSA testing, frequency of primary health care visits, family history of cancer, BMI, smoking alcohol, physical activity, lower urinary tract symptoms, vasectomy, erectile dysfunction, medications for BPH and medications for diabetes, unless variable is the exposure of interest.

^2^variables used in the fully adjusted model.

^3^ BC=breast cancer and OvC=ovarian cancer

^4^P-values are for tests of HR equality between PC stage excluding the HRs of missing value categories.

^5^ Family history of prostate cancer was not adjusted for family history of cancer; height was not adjusted for BMI;

^6^Information obtained from PBS records before PC diagnosis or censoring date.
