# Supplementary material for: Family history, obesity, urological factors and diabetic medications and their associations with risk of prostate cancer diagnosis in a large prospective study
Source: Br J Cancer. 2022 May 24;127(4):735–46. doi: 10.1038/s41416-022-01827-1 (PMC9381576; doi:10.1038/s41416-022-01827-1)
Supplement: Supplementary file 3 — Legends for Supplementary Figures and Tables [file 41416_2022_1827_MOESM3_ESM.docx]

**Supplementary** **Figure and Supplementary** **Table legends**

Supplementary Figure 1 Selection of 45 and Up study participants excluding the first year of follow-up for all participants after the date of enrolment in the 45 and Up Study (n=106,996).

Supplementary Figure 2 Risk for prostate cancer in categories of personal and behavioural characteristics after excluding the first year of follow-up for all participants after the date of enrolment in the 45 and Up Study (n=106,996).

Supplementary Figure 3 Risk for prostate cancer in categories of health-related factors after excluding the first year of follow-up for all participants after the date of enrolment in the 45 and Up Study (n=106,996).

Supplementary Figure 4 Selection of 45 and Up study participants excluding high PSA testers in the 45 and Up Study (n=102,542).

Supplementary Table 1- Characteristics of NSW 45 and Up Study male participants and prostate cancer cases diagnosed in them between study entry and December 2013, after excluding the first year of follow-up for all participants after the date of enrolment in the 45 and Up Study.

Supplementary Table 2- Hazard ratios (HR) and 95% CI for diagnosis of prostate cancer with various sociodemographic and health-related characteristics, after excluding the first year of follow-up for all participants after the date of enrolment in the 45 and Up Study (n=106,996).

Supplementary Table 3- Joint Cox regression (95% CI) for prostate cancer diagnosis with personal, behavioural and health-related factors, after excluding the first year of follow-up for all participants after the date of enrolment in the 45 and Up Study (n=106,996).

Supplementary Table 4- Hazard ratios (HR) and 95% CI for diagnosis of prostate cancer and sociodemographic and health-related characteristics, after excluding high PSA testers in the 45 and Up Study (n=102,542).

Supplementary Table 5- Joint Cox regression (HR, 95% CI) for prostate cancer diagnosis and personal, behavioural and health-related factors, excluding high PSA testers in the 45 and Up Study (n=102,542).

Supplementary Table 6 Joint Cox regression (HR,95% CI) for prostate cancer diagnosis and personal, behavioural and health-related factors for 45 and Up Study male participants including the unknowns (n=107,706).
